# The gut-lung axis: Gut microbiota changes associated with pulmonary fibrosis in mouse models induced by bleomycin

**DOI:** 10.3389/fphar.2022.985223

**Published:** 2022-09-30

**Authors:** Yunyun Quan, Zhujun Yin, Shilong Chen, Jirui Lang, Liyang Han, Jing Yi, Lu Zhang, Qianhua Yue, Weiwei Tian, Ping Chen, Shenglin Du, Jianbo Wang, Ying Dai, Hua Hua, Jin Zeng, Li Li, Junning Zhao

**Affiliations:** ^1^ Department of Pharmacognosy, West China School of Pharmacy, Sichuan University, Chengdu, China; ^2^ Translational Chinese Medicine Key Laboratory of Sichuan Province, Sichuan Academy of Chinese Medicine Sciences, Sichuan Institute for Translational Chinese Medicine, Chengdu, Sichuan, China

**Keywords:** pulmonary fibrosis, gut-lung axis, bleomycin, gut microbiota, signaling pathway

## Abstract

The main objective of this study was to investigate the alterations in the gut microbiota (GM) of pulmonary fibrosis (PF) mice induced by bleomycin (BLM) with its underlying mechanisms. BLM was docked with the targets of TGF-β/SMAD and caspase-3 pathways using the molecular docking technique. HE staining and Masson staining were applied to observe the histopathological changes in the pulmonary tissues. Detection of the apoptotic signals was conducted by flow cytometry and TUNEL staining. The mRNA expression of targets involved in the TGF-β/SMAD and caspase-3 signaling pathways in lungs was determined by qPCR. Immunohistochemistry (IHC) assay was used to detect the expression levels of cleaved caspase-3 and BAX proteins in mice lung tissues. 16S rDNA sequencing analysis was used to investigate the changes of GM in the fecal samples of mice in each group. The results showed that the apoptosis rate of pulmonary cells in the BLM group distinctly increased, with the expression levels of crucial target pro-apoptotic gene *caspase-3*, *BAX* with the corresponding protein, cleaved caspase-3, BAX were apparently elevated. This was accompanied by a significant increase in pro-fibrotic targets level such as *TGF-β, fibronectin, collagen I,* and *collagen III*. The mechanisms of PF induced by BLM were related to apoptosis of lung tissue cells such as alveolar epithelial cells and destroyed alveolar structure and excessive production of extracellular matrix (ECM), which may be bound up with activating TGF-β/SMAD and caspase-3 pathways. As for the GM, it was found that, after BLM induced PF in mice, the micro ecological balance of the GM was destroyed; the distance of PCo1 and Pco2 was significantly elongated, and the relative abundance of some intestinal probiotics like *Catenibacterium* and *Lactobacillus* (*L. johnsonii* and *L. gasseri*) dramatically lowered while the relative abundance of *Verrucomicrobiales* and *Enterobacteriales* substantially increased. Therefore, GM changes associated with PF in mouse models induced by BLM and the concept of “gut-lung axis” might provide an optional therapeutic strategy for PF.

## 1 Introduction

The gut microbiota (GM) of the human body is a complex ecosystem, abundant in total quantity and species, with more than 1,000 kinds of microorganisms—including bacteria, fungi, archaea, and viruses—which is often considered a powerful “organ”. A dynamic balance exists between the host and the GM, preventing the occurrence of disease; if this balance is disturbed, many diseases will manifest (Espirito Santo et al., 2021). Research shows that the influence of the GM on its host includes immunological functions, physiological processes, material metabolism, nutritional transformation, inflammatory reactions, and cell aging (Georgiou et al., 2021). The GM has a close relationship with Parkinson’s, diabetes mellitus, Alzheimer’s, hypertension, non-alcoholic fatty liver disease, atherosclerosis, obesity, chronic lung disease, among others (Chen et al., 2021; Marsland et al., 2015). Although the gastrointestinal and respiratory tracts are considerably separated, respiratory tract epithelium and digestive tract epithelium originate from a common endoderm and both are exposed to the outside, indicating a particular internal relationship between them. Recently, the links between the GM and lung disease have begun to be explored. *Science* magazine published a critical study in 2018 which verified lung inflammation originating in the gut (Mjösberg and Rao., 2018). Thus, a cross-talk exists between the lungs and the GM, termed the “gut-lung axis.” An imbalance in the GM often accompanies many lung diseases, variously affecting their occurrence and progression through the gut-lung axis (Budden et al., 2017). So far, changes in the GM have been shown to be related to asthma (Russell et al., 2013), pneumonia (Schuijt et al., 2016), lung cancer (Zhang J. et al., 2018; Zhuang et al., 2019), COPD, and cystic fibrosis (Chen 2020; Chunxi et al., 2020; Zhang et al., 2020). Some gut microbiomes may therefore be used as biomarkers of lung diseases for therapeutic targeting or to explain the pathophysiology of pulmonary diseases. For example, a study has confirmed that *Bifidobacterium breve* and *Lactobacillus rhamnosus* supplements to COPD-affected mice can reduce alveolar injury and inflammation of the airway (Verheijden et al., 2011).

**FIGURE 1:**
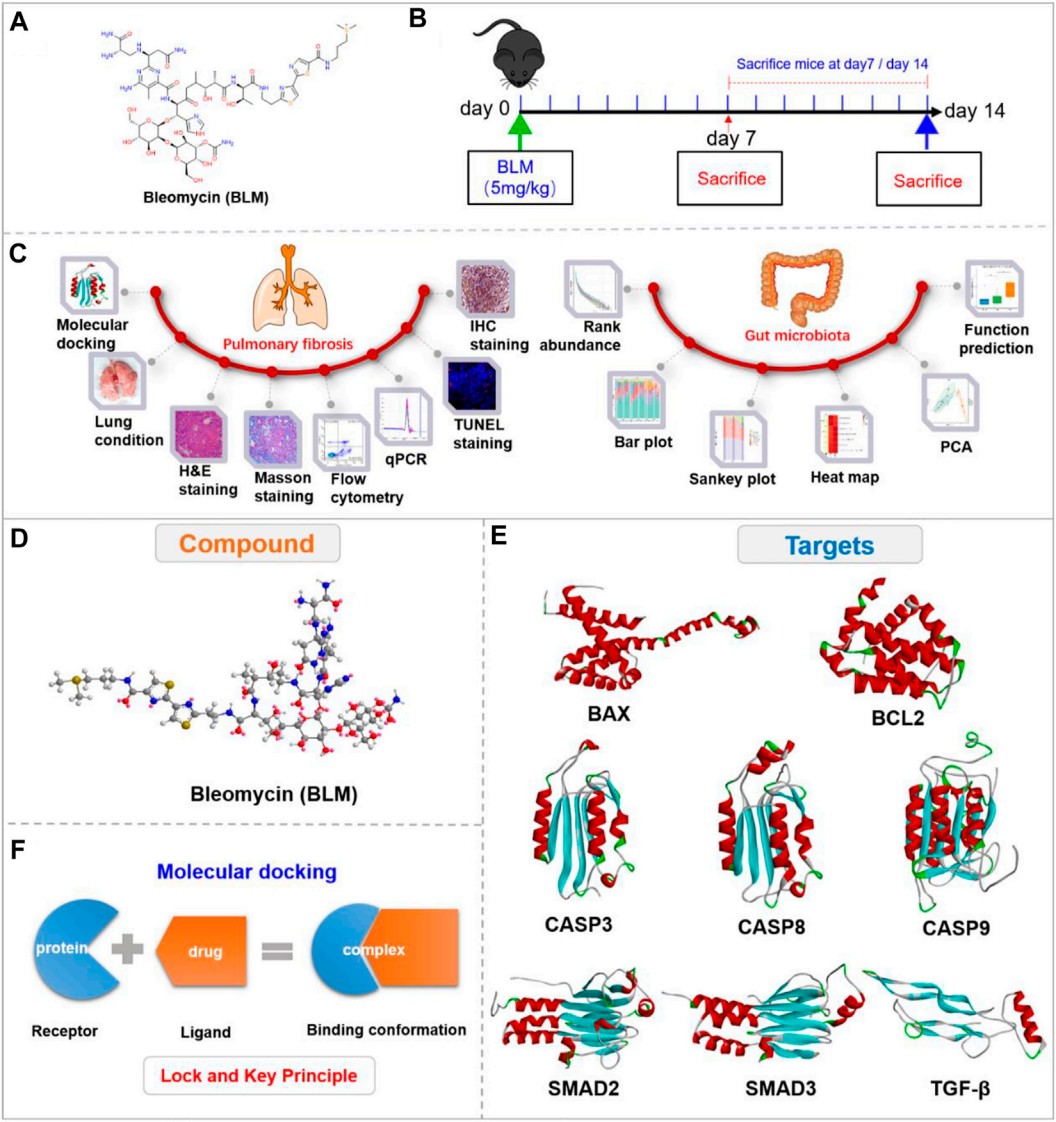
Experimental outline and 3D view of compound with targets. **(A)** Chemical structure of BLM. **(B)** Description of the experimental design. **(C)** Experimental process. **(D)** 3D view of compound BLM. **(E)** The main targets in related signaling pathways. **(F)** The “lock and key principle” of molecular docking.

Pulmonary fibrosis (PF) is the primary pathological process of many chronic lung diseases; it is characterized by abnormal interstitial and alveolar inflammation. An abnormal proliferation of fibroblasts and myofibroblasts leads to an excess of them and deposition of excessive extracellular matrix (ECM) components, destroying the alveolar structure, causing gas exchange dysfunction, and eventually leading to respiratory failure and death (Richeldi et al., 2017; Lederer and Martinez., 2018). The clinical symptoms of PF are progressive dyspnea, dry cough, and fatigue. The risk factors for PF include allergens, chemicals, radiation, smoking, environmental particles, pulmonary infection (virus and bacteria), and drug therapy (Wilson and Wynn, 2009; Reyfman et al., 2018). A number of methods can be applied to construct PF models *in vivo*, such as silica (SiO_2_), bleomycin (BLM), radiation, and paraquat; among these, the pathological changes of the model induced by BLM through intratracheal administration are the most similar to idiopathic PF of humans. Therefore, BLM (C55H84N17O21S3, 1,415.56 g/mol) (Figure 1A) induced PF is a classic model of PF. A correlation between PF and GM has been gradually proposed. Two studies identified a GM disorder in PF mice induced by X-ray thorax irradiation (Li et al., 2020) and PF induced by silica in patients (Zhou et al., 2019). Therefore, the alteration of GM with PF induced by BLM is significant but still needs clarification.

BLM is a broad-spectrum anticancer drug. Its antitumor mechanisms are mainly due to its effect on DNA and combination with the DNA helix, resulting in strand breaks. BLM can chelate metal ions and the attack process may be performed by free radicals produced by the reaction of metal ions with oxygen (activated BLM). Thus, the damaged DNA causes influences synthesis of RNA and protein to some extent, inducing the apoptosis of tumor cells (John ennett et al., 1979; Petering et al., 1990; Hecht, 2000; Saitta et al., 2008). BLM may be especially significant in lung tissue and may also damage alveolar cells following PF. Studies have confirmed that cell death in PF was characterized by apoptosis after epitheliums injury, which plays a significant role in the occurrence of disease (UhalJoshi et al., 1998; Hosseinzadeh et al., 2018; Barbas-Filho et al., 2001; Katzen and Beers., 2020; Falfan-Valencia et al., 2005; Sisson et al., 2010). Apoptosis (programmed cell death) is a self-destructive mechanism of cells mediated by the activation of effector caspase-3 (Serrano-Mollar., 2012; Rogers et al., 2017). BAX and Bcl-2 are apoptosis regulators, which also provoke a broad network of apoptotic signaling to conduct the apoptosis process to cell death (Gaumer et al., 2000; David W; Kamp et al., 2013). As a cytokine, TGF- β is a potent pro-fibrosis stimulant and plays a crucial role in the pathological mechanism of PF (Border and Noble., 1994; Pittet et al., 2001; Lee et al., 2006; Carey et al., 2010; Saito et al., 2018). Research has shown that TGF-β can activate and differentiate epitheliums, and proliferate myofibroblasts to produce collagen. The TGF-β/SMAD is currently a known fibrosis pathway (Xu et al., 2009; Chu et al., 2017; Boutanquoi et al., 2020; Frangogiannis., 2020). The pathological mechanisms of PF induced by BLM have not yet been determined. Consequently, we investigated the PF mechanisms of BLM mainly from the caspase-3 apoptosis pathway and TGF-β/SMAD signaling pathways.

The study showed that the C57BL/6-species mice and male-gender mice are susceptible to BLM (Walters and Kleeberger, 2008). Therefore, based on the “gut-lung axis”, this study researched the PF mechanisms of BLM mainly through HE staining, Masson staining, flow cytometry, qPCR, TUNEL staining, and immunohistochemistry (IHC). It then studied the impact on GM related to PF by analyzing the relative abundance of microbiota, community distance, and function prediction with the use of C57BL/6 male mice *in vivo* (Figure 1C). The study aims to provide optional clinical therapeutic strategies for PF.

## 2 Materials and methods

### 2.1 Materials

BLM was purchased from Selleckchem (Houston, Texas, United States). Apoptosis detection kit was collected from Elabscience Biotechnology Co., Ltd. (Wuhan, China). RNA isolation kit was collected from Chengdu Foregene Biotechnology Co., Ltd. (Chengdu, China). Removal kit for genomic DNA and EvaGreen Express qPCR MasterMix-No Dye were obtained from ABM (Vancouver, Canada). Primer sequence synthesis was conducted by Sangon Biotech (Shanghai, China). DNA Microprep Kit and Zymoclean Gel Recovery Kit were obtained from Zymo Research BIOMICS (California, United States). Prep Kit of DNA Library for Illumina was obtained from BioLabs (United States). NovaSeq 6000 SP Reagent Kit V1.5 was obtained from Illumina (California, United States). *In Situ* Cell Death Detection Kit (TMR red fluorescence) was obtained from Roche Diagnostics (Germany). 3,3′-Diaminobenzidine (DAB) kit was collected from MXB biotechnologies (China). Cleaved caspase-3 and BAX antibodies were collected from CST (United States) and Boster (United States), respectively.

### 2.2 Animals

C57BL/6 mice of male gender (20 ± 2 g, body weight) were obtained from Vital River (Beijing, China). 100 C57BL/6 mice were raised under conditions of SPF in a standard animal laboratory (12 h light/12 h dark cycle, 22°C ± 2°C, air humidity 60% ± 10%). They were habituated to the environment for a week and fed in cages, with five to six mice in each cage. This study was ratified by the Ethical Committee of Sichuan Academy of Chinese Medicine Sciences (SYLL (2021)-033).

### 2.3 Databases and tools

RCSB Protein Data Bank (http://www.rcsb.org/pdb), Gold database, SILVA database 132. Chem3D, AutoDock Vina 1.1.2, AutoDock Tools 1.5.6, Pymol 2.4.0, Discovery Studio 3.5 Client, PubChem database (https://pubchem. ncbi.nlm.nih.gov/), Usearch version 7.1, QIIME v1.9.0, LigPlus. SPSS 21.0, ImageJ, GraphPad Prism 8, ChemBioDraw, and Powerpoint.

### 2.4 Compounds and targets management

The 2D structure of BLM was searched and saved in SDF format through the PubChem website. The 3D structure with minimized energy of BLM (Figure 1D) was obtained by Chem-Bio 3D software. The receptor proteins BAX, BCL2, CASP9, TGF-β, SMAD2, CASP8, SMAD3, and CASP3 were retrieved from the RCSB database and saved in PDB format. The proteins were imported into PyMOL software to remove solvent and organics, then saved in PDB format (Figure 1E). The crystal structures of receptor proteins were imported into Auto Dock tools for hydrogenation optimization and charge modification, then saved in PDBQT format. BLM was imported into AutoDock then similarly saved as PDBQT.

### 2.5 Docking box parameter settings

The receptor proteins were processed through Grid Box in AutoDock tools. The spacing parameter was set to 1, and the appropriate spacing for the X, Y, and Z axes was set to let the ligand rotate in the box in its most extended state. The pocket center was set as the binding site center, and the docking box parameter information was saved in grid.gpf format.

### 2.6 Molecular docking

Based on the “lock and key principle” (Figure 1F) (Chen., 2015), molecular docking of receptors and small molecules was conducted by AutoDock Vina software, in which the PDBQT format files of small molecule ligand and protein receptors were run according to the docking box parameter information. The final results were showed in affinity, and a series of docked output files were obtained. Autodock Vina is developed by Scripps Research Institute (Morris et al., 2009) and systematically evaluated affinity by calculation (Gupta et al., 2018), which is a core indicator of molecular docking (Velec et al., 2005; Neudert and Klebe., 2011; Feinstein and Brylinski., 2015). The lower the affinity, the better the combination between the receptor and ligand. Finally, the binding energy value was analyzed, and the conformation with the lowest affinity was selected for subsequent analysis.

### 2.7 Visual analysis of binding conformation

The binding conformations were visually analyzed using PyMOL, Discovery Studio 3.5 Client, and LigPlus software. 3D cartoon structure of binding conformation and the ligand-residue interaction were displayed by PyMOL to reveal the active site. Discovery Studio Visualizer was used to show the binding interface of the conformation and the receptor-ligand interactions by analyzing complexes and monitoring the residue interactions such as H-bond and Pi-bond to show a 2D diagram. The number of H-bonds between the compound and the protein receptor as well as the hydrophobic interaction were also analyzed by LigPlus in a 2D diagram.

### 2.8 Drug administration

After seven days of adaptation, all mice had the same chance to enter each group: a normal (18 animals) group, a sham (18 animals) group, and BLM (64 animals) group. Because of the risk of death, more mice were in the BLM group. Before administration, all mice fasted for 12 h and drank water as usual. Except for the normal group, each group was given 0.3% pentobarbital sodium according to the weight of the mice by intraperitoneal injection. After being anesthetized, the mice were supinely fixed on the experimental table and the neck was sterilized with 75% alcohol after hair removal. An incision was made along the line in the middle of the neck with surgical scissors, and the trachea was exposed when each layer of tissue was slowly and passively separated with tweezers. BLM was dissolved in saline to prepare a 5 mg/ml solution. The BLM solution was then slowly injected into the trachea of each mouse according to the body weight (5 mg/kg) of the BLM group (Ji et al., 2016; Zhang et al., 2020; Zhang W. Q. et al., 2018; Shen et al., 2021; Ji et al., 2013) with 1 ml micro-injector through the gap between the two tracheal cartilage rings. After injection, the mice were quickly set upright and rotated to make BLM evenly distribute in their lungs. Finally, the incision was sutured and disinfected with an iodophor. For the sham group, the trachea was injected with equivalent saline in the same way and the normal group without any disposal. The animals were raised routinely after waking up.

### 2.9 General condition

The mice were weighed and the number of deaths was recorded regularly every other day or every 2 days. The activity state and hair condition of mice were photographed. Before sacrifice, on the 7th and 14th day after administration, the fresh feces of the mice were obtained with a sterile centrifuge tube and immediately placed in liquid nitrogen, then frozen at −80°C. Then, nine mice in each group were randomly killed for lung tissue by cervical dislocation on day 7 and 14 (Figure 1B). The mice were dissected to collect the lungs, which were washed repeatedly with precooled 0.9% normal saline. The wet weight of each of their lungs was weighed and recorded. After the lung tissue was photographed, part of the pulmonary tissue was placed in 4% paraformaldehyde solution (4% PFA), and part of the lung tissue was placed in PBS solution. The remaining lung tissue was placed in the −80°C refrigerator for later use after quick freezing with liquid nitrogen. The formula was applied to calculate the lung coefficient as follows:

$$Lung\;coefficient=\frac{wet\;lung\;weight\left(g\right)}{body\;weight\left(g\right)}\times 100\%$$

### 2.10 Pathological section analysis

The pulmonary tissues were dehydrated, embedded in paraffin, and cut into 3 μm thick slices with a paraffin slicer. They were then stained with HE and Masson, respectively, and the pathomorphological changes in lung tissue were observed under a microscope, including inflammation, fibrosis, and tissue destruction. The pathological score was used for lung histopathological examination, with the level of alveolitis and PF receiving a Szapiel score (Szapiel et al., 1979) graded from 0 to 3. The specific assessment is shown in Table 1. For HE staining, the nucleus is blue and the cytoplasm is red. Masson staining was performed after paraffin sections’ conventional dewaxing in water. The histopathological changes in lung tissue were observed under a microscope with image acquisition and analysis. A blue signal indicated positive staining for collagen; the percentage of blue collagen fibrosis area was quantificationally analyzed with ImageJ software.

**TABLE 1:** Szapiel score for alveolitis and PF.

Staining	Lung pathology	Severity	Description	Score
HE	Alveolitis	None	No alveolitis	**0**
		Mild	Monocyte infiltration leads to thickening of alveolar septum, only limited to localized pleural lesions accounting for less than 20% of the lung, and the alveolar structure is well preserved.	**1**
		Moderate	A more extensive alveolitis involving 20%–50% of the lung but still predominantly pleura.	**2**
		Severe	Diffuse alveolitis, involving more than 50% of the lung, occasionally, consolidation of air spaces by the alveolar monocytes and some areas of hemorrhage in the interstitium and/or alveolus.	**3**
Masson	Lung fibrosis	None	No evidence of fibrosis	**0**
		Mild	Less than 20% of the lung is involved in localized fibrosis. Fibrosis involves the pleura and the interstitium of the subpleural parenchyma, and alveolar structure is distorted to some extent.	**1**
		Moderate	Widespread fibrosis involves 20%–50% of the lung and for fibrotic areas, most of which extend inward from the pleura and remain focal.	**2**
		Severe	Wide range of fibrosis involves more than 50% of the lung. Fusion lesion with extensive disorder of parenchyma structure, including cystic spaces arranged by cuboidal epithelium.	**3**

### 2.11 Flow cytometry

Apoptosis was measured with flow cytometry. The fresh lung tissue of each group was rinsed with PBS and cut into small particles, when collagenase type I was added for digestion in a constant temperature shaker at 37°C. The digestive solution was filtered with a cell strainer (100 μm nylon) and centrifuged at 300 g for 5 min. The supernatant was abandoned to obtain lung tissue cells, then resuspended using PBS. 1–5 × 10^5^ lung tissue cells were collected after centrifugation and removal of the supernatant. Cells were resuspended with 100 μl diluted 1×annexin V binding buffer. Then the staining solution of 2.5 μl annexin V-FITC and 2.5 μl PI was added separately. After gentle vortex mixing, the cells were incubated away from light in room temperature for 15–20 min. Finally, these samples were again mixed using 400 μl binding buffer immediately detected by CytoFLEX flow cytometry (Beckman Coulter, United States). The blank control and single dye tube were used for voltage regulation, compensation regulation, and other settings.

### 2.12 qPCR

Total RNA was obtained using RNA extract reagent from lung tissue to form a solution with 30 μl water of RNase-free. K2800 Nucleic Acid/Protein Analyzer (Beijing Kaiao Technology Development Co., Ltd., China) was applied to measure the RNA purity expressed in the OD value of A260/A280. A total amount of RNA was used to synthesize cDNA according to the following procedures: 25°C for 10 min, 42°C for 15 min, and 85°C for 5 min. In the end, the samples were put on ice for cooling. Then the cDNA was used to conduct a qPCR reaction. The following were the qPCR reaction conditions: pre-denaturation temperature 95°C for 3 min, and then 40 cycles were performed with denatured temperature 95°C for 10 s and annealing temperature 60°C for 30 s. In order to guarantee that a single product was conducted by amplification, the melting curve analysis was carried out after each PCR reaction. The value of Ct was recorded and the 2^−ΔΔCt^ method was applied to compute the target gene’s relative mRNA expression. The primer sequences for qPCR are shown in Table 2.

**TABLE 2:** Primers used for qPCR in this study.

Gene	NCBI gene ID	Direction	Primer sequence (5′-3′)	Product length (bp)	Annealing temperature (°C)
*gapdh*	14433	Forward	GGT​TGT​CTC​CTG​CGA​CTT​CA	183	60
		Reverse	TGG​TCC​AGG​GTT​TCT​TAC​TCC		
*caspase3*	12367	Forward	CTC​GCT​CTG​GTA​CGG​ATG​TG	201	60
		Reverse	TCC​CAT​AAA​TGA​CCC​CTT​CAT​CA		
*caspase8*	12370	Forward	TGC​TTG​GAC​TAC​ATC​CCA​CAC	171	60
		Reverse	GTT​GCA​GTC​TAG​GAA​GTT​GAC​C		
*caspase9*	12371	Forward	AGC​CAG​AGG​TTC​TCA​GAC​CAG	103	60
		Reverse	ATA​TCT​GCA​TGT​CCC​CTG​ATC​T		
*bcl-2*	12043	Forward	GCT​ACC​GTC​GTG​ACT​TCG​C	147	60
		Reverse	CCC​CAC​CGA​ACT​CAA​AGA​AGG		
*bax*	12028	Forward	AGA​CAG​GGG​CCT​TTT​TGC​TAC	137	60
		Reverse	AAT​TCG​CCG​GAG​ACA​CTC​G		
*collagen I*	12842	Forward	TAA​GGG​TCC​CCA​ATG​GTG​AGA	203	60
		Reverse	GGG​TCC​CTC​GAC​TCC​TAC​AT		
*collagen III*	12825	Forward	CTG​TAA​CAT​GGA​AAC​TGG​GGA​AA	144	60
		Reverse	CCA​TAG​CTG​AAC​TGA​AAA​CCA​CC		
*α-SMA*	11475	Forward	CCC​AGA​CAT​CAG​GGA​GTA​ATG​G	104	60
		Reverse	TCT​ATC​GGA​TAC​TTC​AGC​GTC​A		
*fibronectin*	14268	Forward	ATG​TGG​ACC​CCT​CCT​GAT​AGT	124	60
		Reverse	GCC​CAG​TGA​TTT​CAG​CAA​AGG		
*vimentin*	22352	Forward	TCC​ACA​CGC​ACC​TAC​AGT​CT	100	60
		Reverse	CCG​AGG​ACC​GGG​TCA​CAT​A		
*E-cadherin*	12550	Forward	CAG​TTC​CGA​GGT​CTA​CAC​CTT	131	60
		Reverse	TGA​ATC​GGG​AGT​CTT​CCG​AAA​A		
*smad2*	17126	Forward	AAG​CCA​TCA​CCA​CTC​AGA​ATT​G	100	60
		Reverse	CAC​TGA​TCT​ACC​GTA​TTT​GCT​GT		
*smad3*	17127	Forward	CAT​TCC​ATT​CCC​GAG​AAC​ACT​AA	126	60
		Reverse	GCT​GTG​GTT​CAT​CTG​GTG​GT		
*TGF-β*	11461	Forward	CCA​GAT​CCT​GTC​CAA​ACT​AAG​G	169	60
		Reverse	CTC​TTT​AGC​ATA​GTA​GTC​CGC​T		

### 2.13 TUNEL staining

The paraffin slices of the lung tissue with 3 μm thickness were placed into dewaxing liquid I for 15 min, dewaxing liquid II for 15 min, dewaxing liquid III for 15 min, absolute alcohol I for 5 min, absolute alcohol II for 5 min, 85% alcohol for 5 min, 75% alcohol for 5 min, then washed with distilled water. The slices were circled and soaked in TBS. The membrane breaking solution was added dropwise and digested at room temperature for 8 min. The slices were washed with TBS three times, 5 min each time. 10% goat serum was added to block the nonspecific reaction at room temperature for 30 min. The serum was then discarded and TUNEL reaction mixture was added to the slices for incubation of 60 min in the wet box at 37°C to avoid light. DAPI was used to stain nucleus for 10 min. The slices were sealed with anti-fluorescence quenching agent and observed under fluorescence microscope. Photographs were then taken. The nucleus showed blue fluorescence (DAPI) and the TUNEL positive signal showed red fluorescence (TMR).

### 2.14 Immunohistochemistry

Paraffin sections of the lung tissue with 3 μm thickness were dewaxed to conduct antigen retrieval in antigen retrieval buffer. The slices were immersed in 3% H_2_O_2_ for 30 min at room temperature. The slices were circled with an IHC pen and put into TBST, and then 10% goat serum was added for incubation of 30 min at room temperature. Lung tissue slices were incubated overnight with primary antibodies (cleaved caspase-3 and BAX) at 4°C, then incubated with a second antibody for 45 min at 37°C. DAB solution was used for color reaction. Hematoxylin was used to stain the nucleus for 1 min. The slices were observed under the microscope after being sealed, and the images were then collected.

### 2.15 Gut microbiota sequencing analysis

DNA Microprep Kit was used to extract the genomic DNA from the feces in each group, and the gDNA was purified. The gDNA integrity was detected by 0.8% agarose gel electrophoresis, followed by Infinite F200 Microplate Reader (Tecan, Switzerland) for nucleic acid concentration detection. Primers (Table 3) were utilized to amplify the 16S rDNA V4 region of the sample by PCR, each sample being repeated three times. PCR products were mixed from the same sample, which was checked by agarose gel electrophoresis. The products of PCR in the target bands of the qualified samples were recovered and purified by a Zymoclean Gel Recovery Kit and then quantified with Qubit 2.0 (Thermo Fisher Scientific, United States). The library was built with the Prep Kit of DNA Library for Illumina, and high-throughput sequencing was performed using the NovaSeq 6000 SP Reagent Kit V1.5 by the PE250 sequencing method. QIIME v1.9.0 was used to perform quality control, and chimeras were removed using the Uchime algorithm and gold database. Operational taxonomic units (OTU) clustering was performed at a similarity level of 97%. Annotation analysis was performed using UCLUST taxonomy and SILVA database 132. The information abundance of the community was calculated, and community composition analysis, community distance analysis, and community function prediction were conducted.

**TABLE 3:** Primers used for GM sequencing analysis in this study.

Name	Primer sequence
515F	5′-GTGYCAGCMGCCGCGGTAA-3′
806R	5′-GGACTACHVGGGTWTCTAAT-3′

### 2.16 Statistical analysis

SPSS 21.0 software was used to statistically process the data, and the measurement results were expressed in mean ± SD ( ±s ). The difference among groups was performed with one-way ANOVA, and pairwise comparison was conducted with LSD. *p* < 0.05 implied that the difference was statistically significant. PerMANOVA and Anosim with Weighted UniFrac were carried out for the community distance analysis. GraphPad Prism 8, ChemBioDraw, and PowerPoint were used for drawing.

## 3 Results

### 3.1 Affinity between bleomycin and targets in caspase-3 and TGF-β/SMAD signaling pathways

**FIGURE 2:**
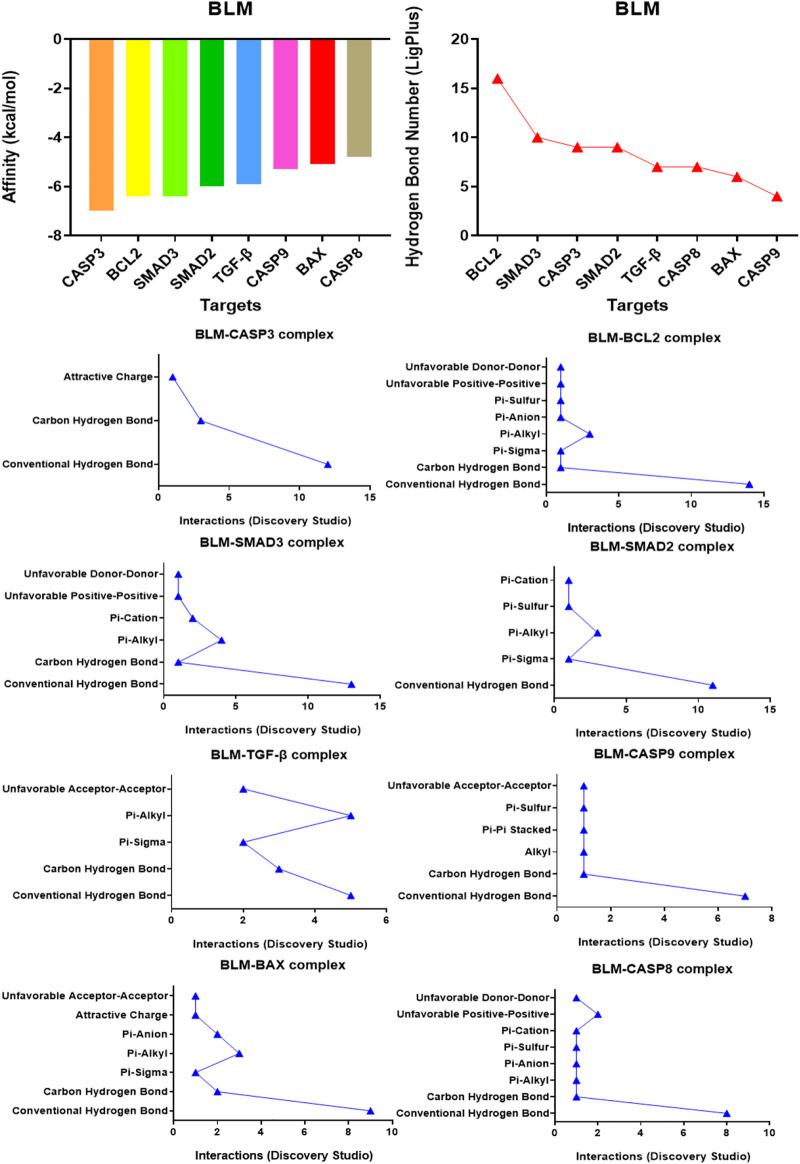
The affinity of BLM binding to each target and the interactions within the complex.

Affinity is the value used by the molecular docking software AutoDock Vina to show the binding ability of small molecules with proteins. The affinity result is the evaluation index: the lower the affinity (binding energy), the stronger the matching binding ability. The results showed that the affinity of BLM binding to all protein targets was ≤0.0 kcal/mol. Among them, target caspase-3 had the best binding effect on BLM (affinity, −7.0 kcal/mol) and target caspase-8 had the weakest binding effect on BLM (affinity, -4.8 kcal/mol) (Figure 2). The results suggested that caspase-3, BCL2, SMAD3, SMAD2, TGF-β, caspase-9, BAX, and caspase-8 were the potential targets of BLM’s PF because of the binding spontaneously. Among them, caspase-3, BCL2, caspase-9, BAX, and caspase-8 are mainly involved in the apoptosis signaling pathways, and SMAD3, SMAD2, and TGF-β are mainly involved in the fibrosis signaling pathway.

### 3.2 Visual results of bleomycin and targets in caspase-3 and TGF-β/SMAD signaling pathways

**FIGURE 3:**
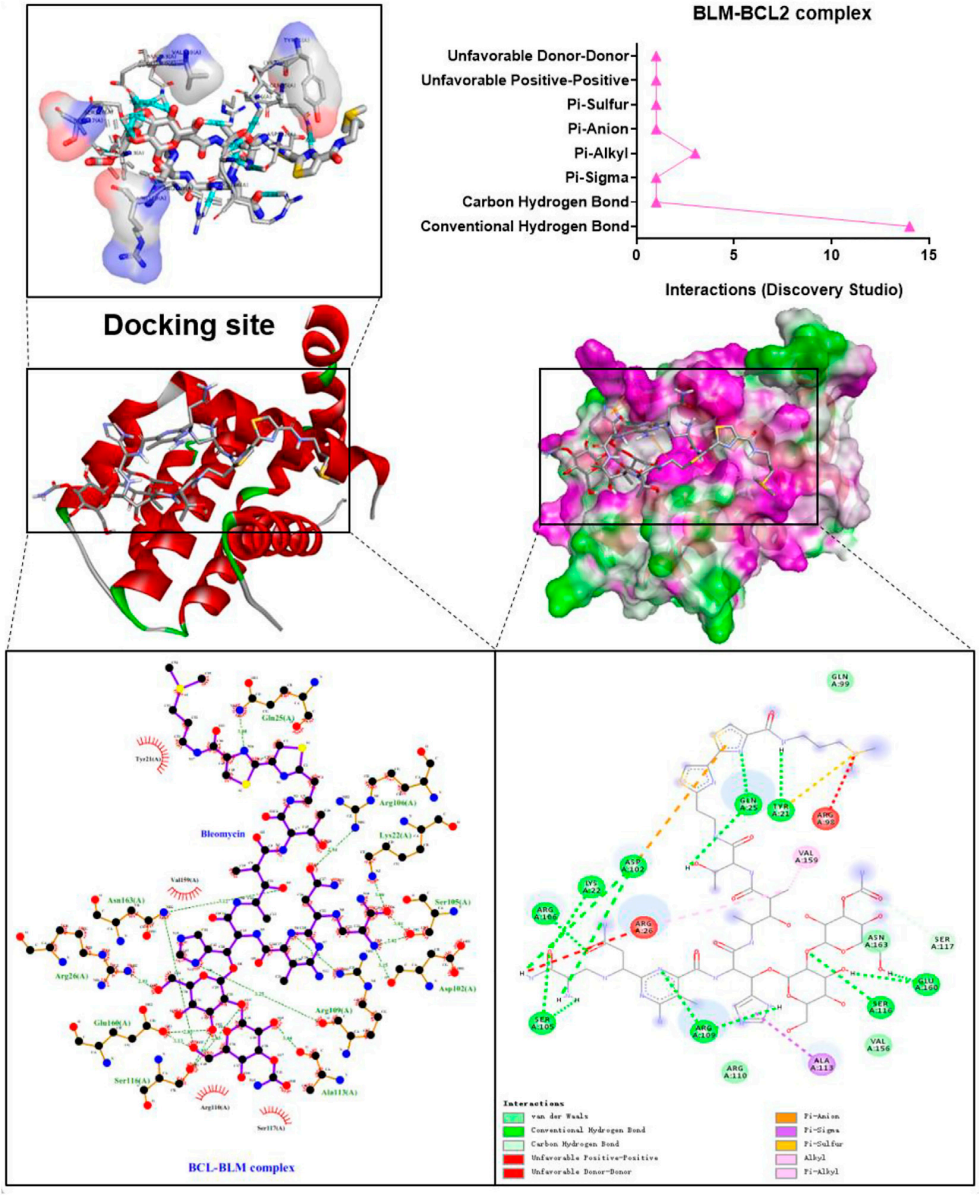
Visual analysis for BLM-BCL2 complex of molecular docking.

The binding confirmation obtained by molecular docking can simulate the binding mode of receptor-ligand complexes to a certain extent, thus providing a scientific basis for the ligand and receptor interaction. Through visual analysis, it was found that BLM could form complex interactions with receptor targets caspase-3, caspase-8, caspase-9, BAX, BCL2, TGF-β, SMAD2, and SMAD3, respectively, including conventional H-bond, carbon H-bond, Pi-sulfur, Pi-cation, Pi-anion, Pi-alkyl, Pi-sigma, Pi-Pi stacked, attractive charge, and hydrophobic. Among these, as for H-bond, the best was the binding conformation of BLM-BCL2, which could form as many as 15 H-bonds (the H-bond number was 16 as analyzed by LigPlus while it was 15 when analyzed by Discovery Studio) (Figure 2). The top-four targets were caspase-3, BCL2, SMAD2, and SMAD3, which can better match with BLM, through the comprehensive comparison of affinity and the number of H-bonds. On the one hand, the top-four targets can bind to BLM with affinity ≤ −6.0 kcal/mol, while, on the other hand, they can form H-bonds with number≥ 9 (by LigPlus) and with number>10 (by Discovery Studio) (Figure 2). The example of visual analysis for BLM-BCL2 complex of molecular docking was shown in (Figure 3)—the others were shown in Supplementary Figures S1–S7). Although the docking effect of caspase-8, caspase-9, BAX, and TGF-β with BLM is weaker than caspase-3, BCL2, SMAD2, and SMAD3 with BLM, they can still bind spontaneously to form complex interactions, which is also worthy of verification by animal experiments. This suggests that the PF mechanism of BLM may theoretically be associated with caspase-3, caspase-8, caspase-9, BAX, BCL2, TGF-β, SMAD2, and SMAD3 through virtual analysis by molecular docking.

### 3.3 Lung tissues, percent survival, body weight, and lung coefficient changes

**FIGURE 4:**
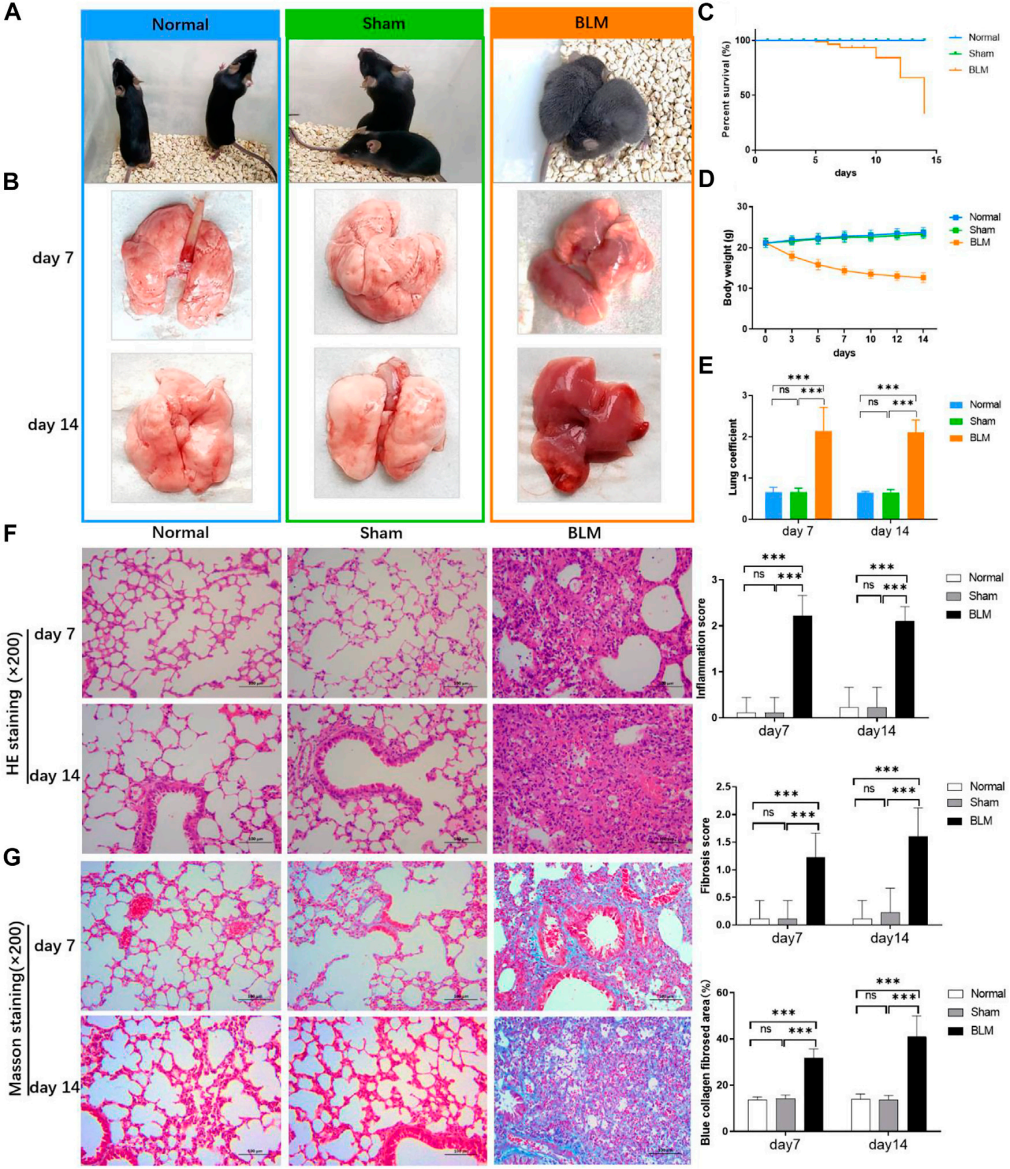
Effect of BLM on lungs, percent survival, body weight and lung coefficient of mice and the pathological section analysis of HE and Masson staining. **(A)** Active state of mice. **(B)** The lung tissues of mice. **(C)** Percent survival. **(D)** Body weight changes. **(E)** Lung coefficient changes. **(F)** HE staining with magnification times (×200). **(G)** Masson staining with magnification times (×200). ***p < 0.001 *vs*. normal and sham group. “ns” indicates not significant.

The mice in the normal and sham groups were in good condition, with markedly increased weight, usual activities, good appetite, and smooth, shiny hair. The mice in the BLM group were in poor condition and curled up, hunched back with a thin body, less activity, no diet and drinking, shortness of breath, and messy, rough, dull hair (Figure 4A). Both sham and normal groups of mice survived with no death within 14 days, and the survival rate of both groups was 100.00%. From day 5, mice in the BLM group began to die. A total of 15 mice died until day 7, and another 30 mice died from day 8 to 14. Up to day 7, overall mortality was 23.4% and the survival rate was 76.6%. Up to day 14, overall mortality was 75% and the survival rate was 25% (Figure 4C). The weight of the mice in the sham and normal groups gradually increased and the mice weight of BLM continuously decreased. The weight loss was rapid in the first seven days, and slowed in the next seven days (Figure 4D). As indicated in Figure 4E, there was no apparent discrepancy in lung coefficient between the sham and normal groups. The lung coefficient of the BLM group was prominently higher than the other two groups on both day 7 and 14. The pulmonary tissues in the normal and sham groups were normal, light in mass, and showed pink and white color with a clear soft texture, smooth surface, and good elasticity. In the BLM group, there were lesions in lung tissues with heavy mass, dark red color, substantial changes, fibrosis, and hard texture (Figure 4B).

### 3.4 Lung tissue inflammation and fibrosis by HE and Masson staining

As seen in Figures 4F,G, the results of HE pathological section analysis indicated that the lung tissue of the normal and sham groups were in normal condition, the alveolar cavity structure was intact, and the alveolar epithelium and capillaries were normal. In the BLM group, the lung tissue showed lesions, prominent fibrosis, a damaged or collapsed alveolar wall, severe alveolar structure disorder, interstitial infiltration of inflammatory cells, increased connective tissue in the lungs, and increased alveolar cavity fusion. No noticeable difference was demonstrated in the sham and normal groups’ inflammation score, fibrosis score, and blue collagen fibrosis area. Compared with the other two groups, BLM distinctly increased inflammation scores on day 7 and 14. For Masson staining (the blue area is collagen fiber), collagen barely existed in the pulmonary tissues of the normal and sham groups. In the BLM group, there were large blue areas in the interstitial area of the lung. The lung tissue of the BLM group showed alveolar cavity collapse and deformation, interval widening, and extensive collagen deposition. BLM promoted fibrotic lesions (fibrosis score and blue collagen fibrosis area) in the mice on day 7 and 14.

### 3.5 The apoptosis rate of lung tissue cells

The results of annexin V-FITC/PI two-color showed that the distribution of each cell population accorded with its detection principle. As seen in Figure 9A, Q1-LL indicated normal cells; Q1-LR (annexin V ^+^ PI^-^) indicated cells in early apoptosis; Q1-UR (annexin V ^+^ PI^+^) indicated late apoptotic cells; Q1-UL (annexin V^-^PI^+^) indicated necrotic cells. The results showed that the normal group was not distinct from the sham group in the apoptotic cell rate. Compared with the normal and sham groups, the total apoptosis rate of mice lung tissue cells in the BLM group was conspicuously increased on day 7 and 14.

### 3.6 The mRNA expression level of targets in caspase-3 and TGF-β/SMAD pathways

**FIGURE 5:**
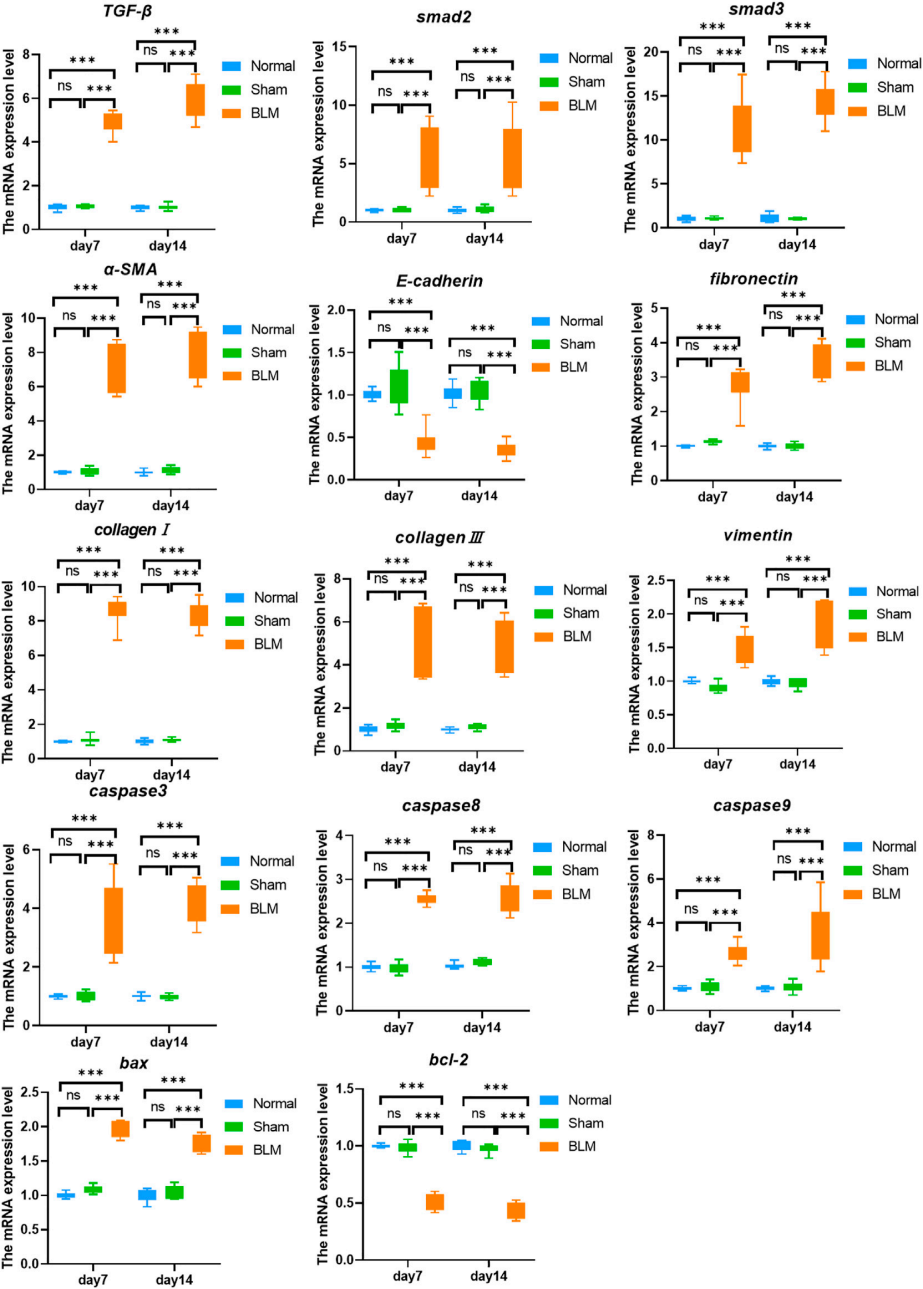
The mRNA expression level of targets in caspase-3 and TGF-β/SMAD pathways. ***p < 0.001 *vs*. normal and sham group. “ns” indicates not significant.

As indicated in Figure 5, at the mRNA level, the normal group existed with no distinct differentiation from the sham group. The results showed that the expression levels of target genes *TGF-β, smad2, smad3, α-SMA, fibronectin, vimentin, collagen I, collagen III* of TGF-β/SMAD pathways were notably increased at day 7 and 14 after injection with BLM. Similarly, the target gene *BAX, caspase-9, caspase-3, and caspase-8* mRNA expression levels in the apoptotic pathway were notably increased on day 7 and 14 and remarkably higher than the normal and the sham groups. The target gene *E-cadherin* and the apoptosis inhibitory gene *bcl-2* mRNA expression level of the BLM group were substantially reduced than that in the normal and sham groups.

### 3.7 The effect of bleomycin on apoptosis in lung tissue

**FIGURE 6:**
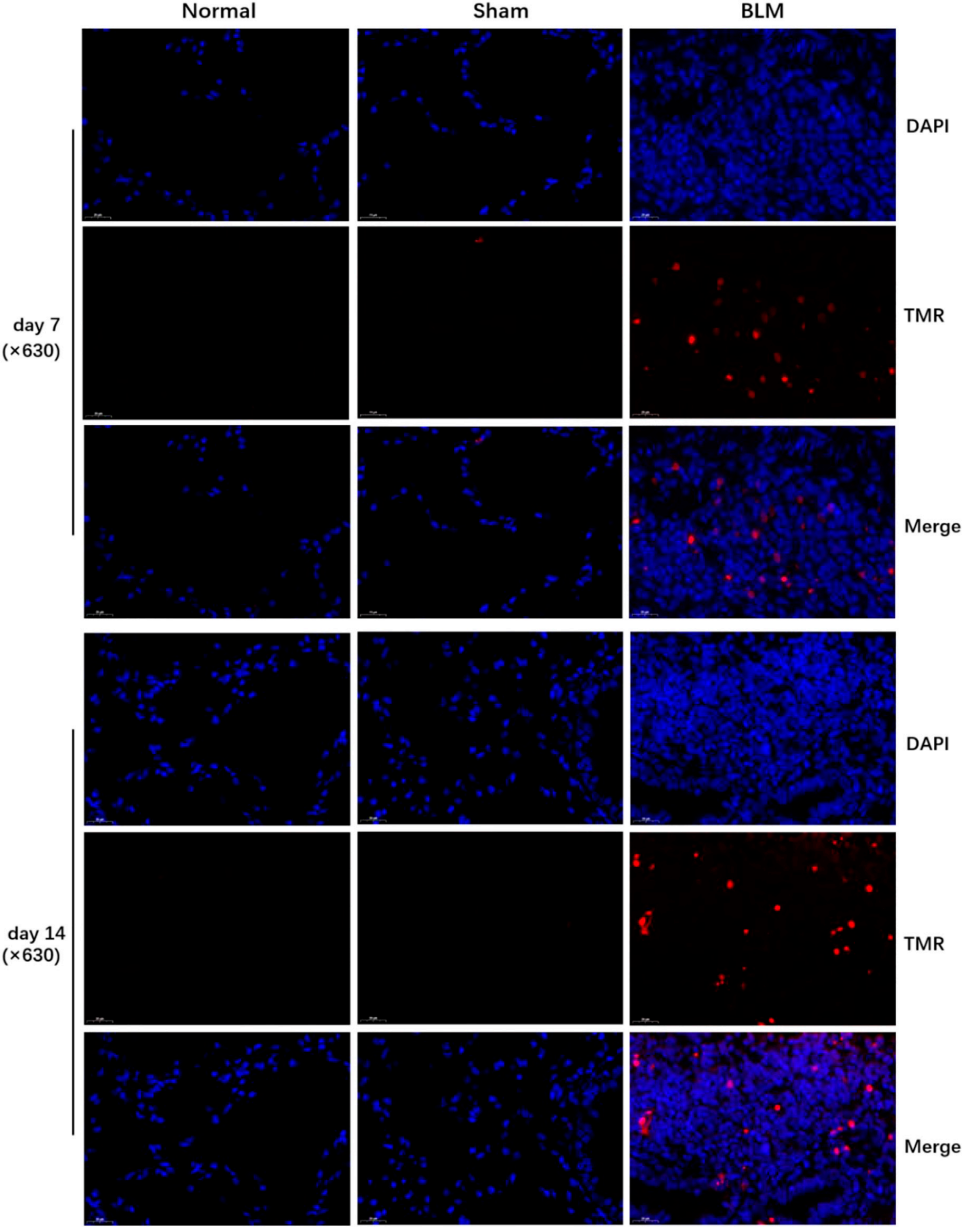
TUNEL assay of mice lung tissues (×630). DAPI (*blue*), TMR(*red*).

**FIGURE 7:**
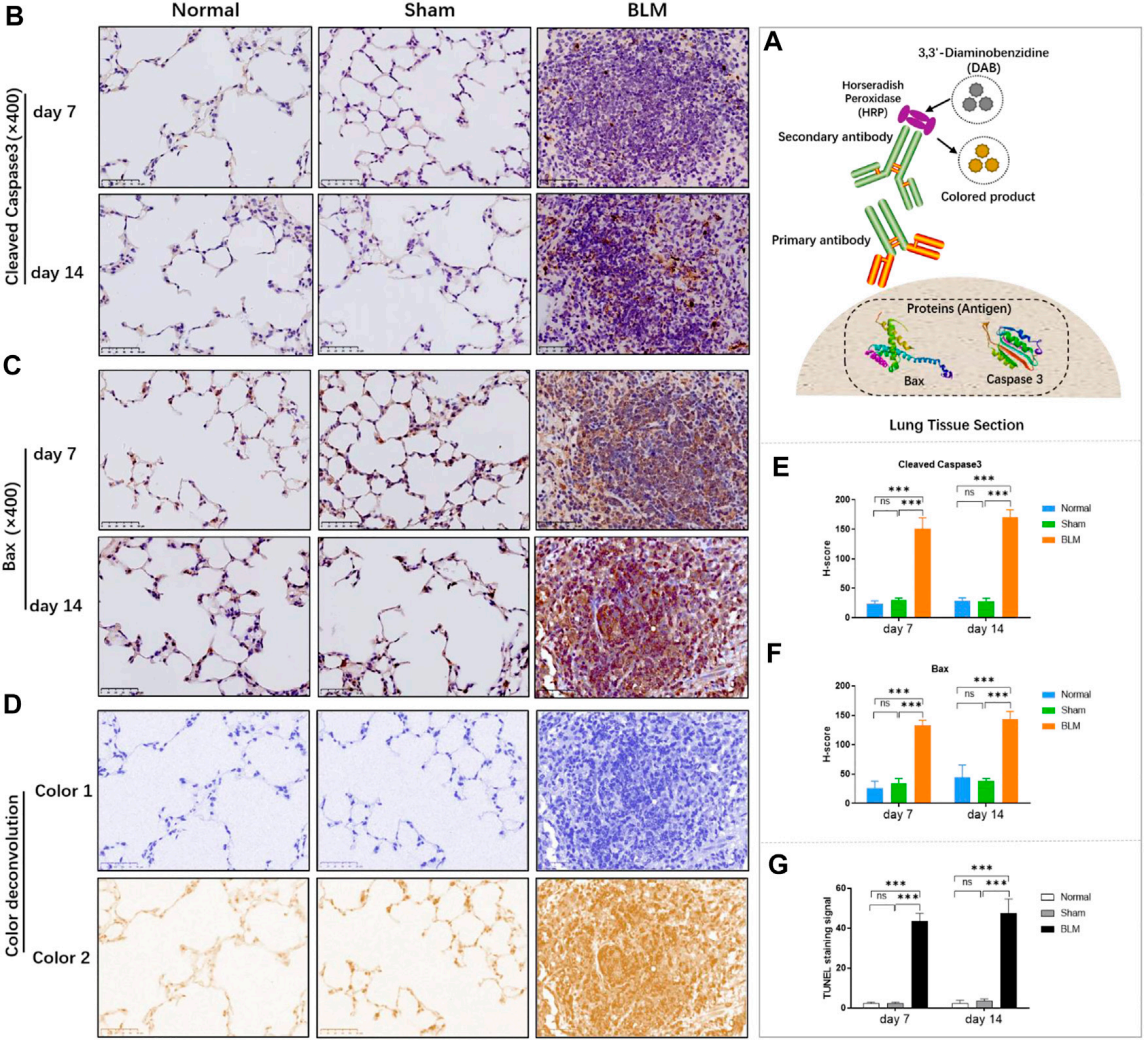
The effect of BLM on the expression of pro-apoptotic key proteins in lung tissue of mice and the quantitative results of TUNEL apoptosis assay. **(A)** IHC schematic diagram. **(B)** IHC staining of cleaved caspase-3 in lung tissue (×400). **(C)** IHC staining of BAX in lung tissue (×400). **(D)** Examples of color deconvolution analysis. **(E)** Quantitative results of cleaved caspase-3 in lung tissue. **(F)** Quantitative results of BAX in lung tissue. **(G)** Quantitative results of TUNEL apoptosis in lung tissue. ***p < 0.001 *vs*. normal and sham group. “ns” indicates not significant.

According to Roche’s TUNEL apoptosis detection kit, the apoptotic signals were detected as red fluorescence under the microscope. TUNEL staining demonstrated that the alveolar structure of lung tissue in the normal and sham groups was intact and that there were few red fluorescent signals in the sham and normal groups. Compared with the normal and sham groups, a large number of dense red apoptotic signals of lung tissue were observed in the BLM group (Figure 6); the alveolar structure of lung tissue in the BLM group was severely damaged, accompanied by evidently increased apoptotic signals on day 7 and 14, mainly distributing in alveolar epithelium (Figure 7G).

### 3.8 The effect of bleomycin on the expression of pro-apoptotic key proteins

Applying antigen-antibody reaction and color products, IHC can directly show the distribution of target protein (antigen) in tissue cells through localization, and qualitative and relative quantitative analysis (Figure 7A). Caspases, a family of cysteine proteases, are the key mediators of programmed cell death or apoptosis. Cleaved caspase-3 is the activated form of caspase-3. BAX is also the apoptosis regulator, which provokes a broad network of apoptotic signaling to conduct the apoptosis process to cell death. Hence, in order to further verify the results of molecular docking and qPCR, the expression of pro-apoptotic key proteins, cleaved caspase-3 and BAX, in mice lung tissue was investigated by IHC. In the stained image, the colored product (HRP-DAB) is displayed in brownish yellow. The more targeted protein expression, the more the colored product deposit through the color deconvolution analysis (Figure 7D). The results identified that the alveolar structure of the normal and sham groups was in good condition, accompanied by a small amount of protein expression of cleaved caspase-3 and BAX (Figures 7B,C). The alveolar structure of the BLM group was destroyed with a great deal of pro-apoptotic protein expression in lung tissues. The quantitative results revealed that there was no significant difference between the sham and normal groups. Compared with the normal group, the expression of cleaved caspase-3 and BAX proteins in the lung tissues of the BLM group significantly increased on day 7 and 14 (Figures 7E,F).

### 3.9 Alterations in the gut microbiota of pulmonary fibrosis mice induced by bleomycin

#### 3.9.1 Bleomycin can change the community abundance of gut microbiota at order, phylum, family, genus, and species levels

**FIGURE 8:**
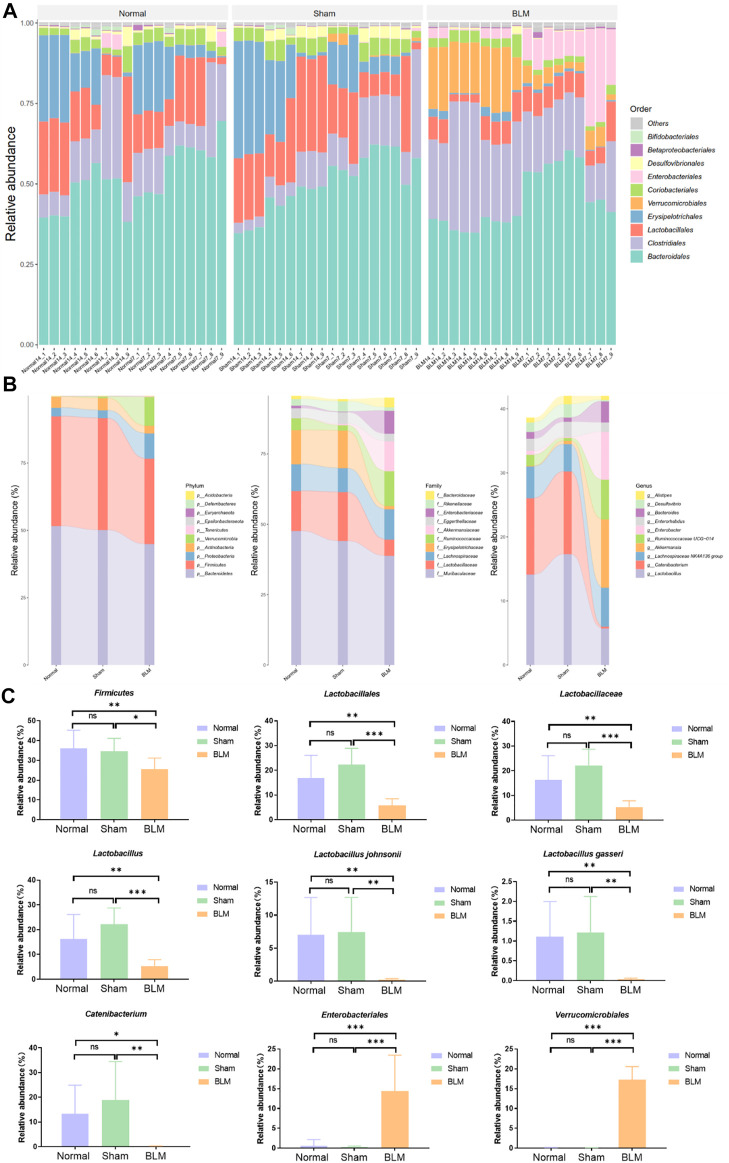
Community composition and difference analysis of GM. **(A)** Bar plot of relative abundance at order level. **(B)** Sankey plot of relative abundance at phylum, family, and genus level. **(C)** Quantitative analysis of representative community. *p < 0.05, **p < 0.01, ***p < 0.001 *vs* normal and sham group. “ns” indicates not significant.

**FIGURE 9:**
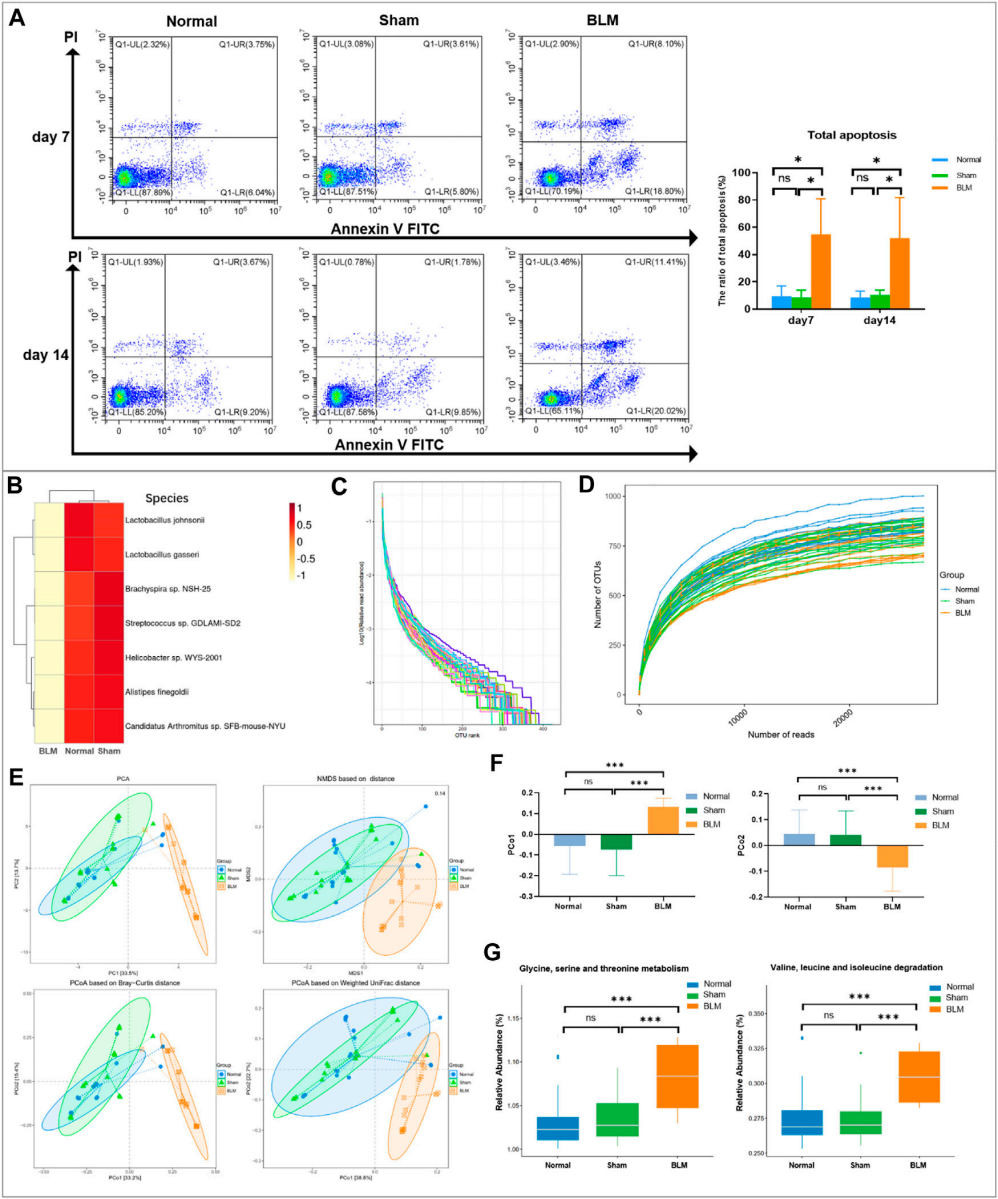
Apoptosis analysis by flow cytometry and GM sequencing analysis. **(A)** Apoptosis analysis by flow cytometry. **(B)** Cluster heatmap analysis of high abundance at species level. **(C)** Rank abundance curve. **(D)** Rarefaction curve. **(E)** Community distance analysis. **(F)** Distance of PCo1 and PCo2. **(G)** Community function prediction. *p < 0.05, ***p < 0.001 *vs* normal and sham group. “ns” indicates not significant.

In order to intuitively view and compare the communities with high abundance and their proportion at different classification levels, the top 10 at the order level were selected to generate a bar plot of relative abundance (Figure 8A). The top 10 at the phylum, family, and genus levels were selected to generate a Sankey plot (Figure 8B). The top-seven at the species level with abundance were selected to generate a heat map and each line was standardized by z-score. (Figure 9B). The results showed that BLM could alter the relative abundance of some microbiota. For example, there was a remarkably decreased relative abundance of *Firmicutes* (day 7), *Lactobacillales* (day 14), *Lactobacillaceae* (day 14), *Lactobacillus* (day 14), *Catenibacterium* (day 14), *Lactobacillus gasseri* (day 14), and *Lactobacillus johnsonii* (day 14), and a dramatically increased relative abundance of *Verrucomicrobiales* (day 14) and *Enterobacteriales* (day 7) in the BLM group (Figure 8C). The above showed that BLM can change the community abundance of many microbiotas in mice gut.

#### 3.9.2 The species abundance and community evenness of gut microbiota

The rank abundance curve can explain species abundance and community evenness. The range on the horizontal axis of each group in this study was extensive and decreased gently, indicating that the species abundance of GM in each group was high and the species distribution was uniform. As shown in Figure 9C, the curve of all samples in this study tended smooth, illustrating that all data in each group met the sequencing requirements and that the microbial diversity information of each group can be reflected comprehensively. By drawing the rarefaction curve, species richness in the sample can be indirectly reflected. As shown in Figure 9D, the rarefaction curve tendency was flat, which indicated that the sequencing process embraced almost all the species in each group of this study.

#### 3.9.3 Bleomycin can increase the distance of PCo1 and PCo2 of gut microbiota

To obtain the degree of difference among the three groups, common beta diversity measures are used to calculate the distance between two samples. The higher the community composition similarity between groups, the closer their distance is. Non-metric multi-dimensional scaling (NMDS), principal coordinates analysis (PCoA), and principal component analysis (PCA) are common methods for analyzing beta diversity. Community distance analysis of each group is depicted in Figure 9E. The results displayed a large overlap between the normal and sham groups, with a prominent separation between the BLM and normal mice as well as the sham group. The distance of PCo1 and PCo2 showed no marked discrepancy in the distance between the normal and sham groups. However, there arose noteworthy differences in the distance between the BLM group and the normal ones and the sham group, respectively (Figure 9F). This suggests that BLM markedly increased the distance of PCo1 and PCo2 and had a direct influence on the beta diversity of GM.

#### 3.9.4 Bleomycin can change the metabolism and degradation of amino acids

The functional prediction results were enriched and depicted in Figure 9G. The results showed that, in the prediction of metabolic function, in comparison with normal and sham groups, BLM dramatically increased the valine, leucine, and isoleucine degradation and the glycine, serine, and threonine metabolism of the GM. A study identified that fasting could activate the serine-glycine-threonine metabolic axis (Aon et al., 2020). Consequently, the damage of BLM to mouse lung tissue resulted in no diet with thin mice bodies, thus increasing the glycine-serine-threonine metabolism. Branched-chain amino acids (BCAAs) are the general name of leucine, valine, and isoleucine, which are the base for all life forms. Therefore, BLM may accelerate the essential amino acid degradation to affect the health of mice through altering the GM.

## 4 Discussion

BLM conducts potent and broad anticancer activities against squamous cell carcinomas, testicular cancer, malignant lymphomas, cervical cancer, ovarian cancer, sarcomas, and melanomas (Lazo et al., 1990; Chen and Stubbe., 2005; Galm et al., 2005). However, it has serious side effects, among which pulmonary toxicity is the most significant. Therefore, investigating the underlying molecular mechanism of this side effect of BLM will help improve the clinical application of BLM for cancerous remedies. The pulmonary toxicity of BLM induction is similar to human idiopathic PF, of which the pathogenesis has not yet been determined. Therefore, the pulmonary toxicity mechanisms of BLM were studied here to provide a reference concerning research into anti-PF drugs. The antitumor mechanism of BLM is mainly owing to the effect on DNA and combination with the DNA helix resulting in strand breaks that induce cancer cell apoptosis. Hence, we speculated that BLM may also have toxic effects on lung tissue cells. The mechanisms of its toxic or side effects on normal lung tissue cells may be similar or dissimilar to cancer cells. It was reported that one of the pathogenic mechanisms of PF was epithelial apoptosis (King et al., 2011; Martinez et al., 2017; Richeldi et al., 2017), which was related to the caspase-3 apoptosis signaling pathway (Kasper and Barth, 2017). Molecular docking research is a method of studying the interactions and predicting the affinity and binding conformation between small molecule ligands and biomacromolecule receptors by using mathematical, biological, and computer models; these can help us better understand the complexity of life systems (Ciemny et al., 2018; Brodl et al., 2018; Sun et al., 2010). Therefore, in this study, we first used molecular docking to comprehensively analyze the effect and influence of BLM on the possible regulatory apoptosis and fibrosis signaling pathways to elucidate the molecular drug mechanisms.

**FIGURE 10:**
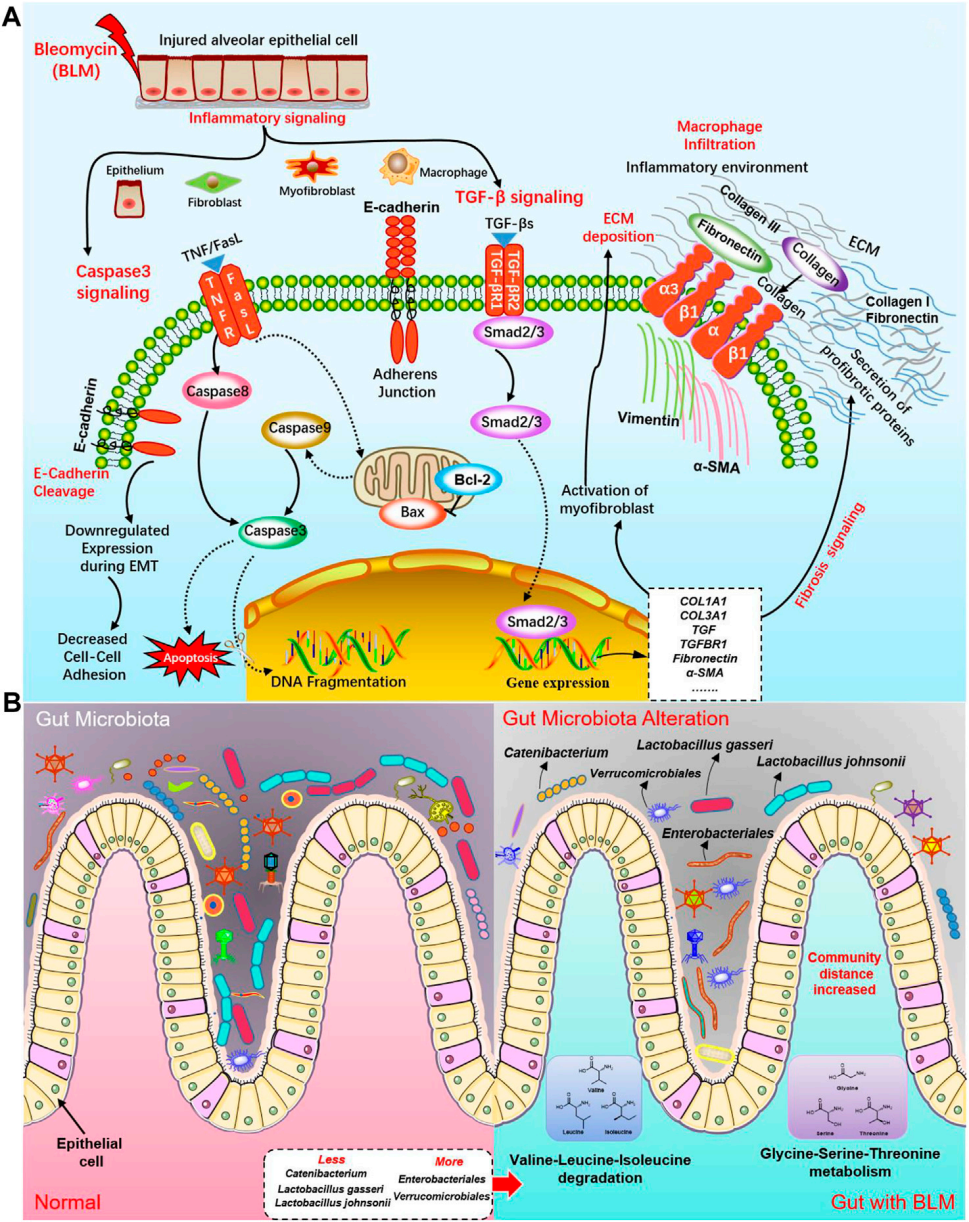
The potential molecular mechanisms of BLM on PF and the influence on GM. **(A)** The potential molecular mechanisms of BLM on PF in caspase-3 apoptosis signaling pathway and TGF-β/SMAD fibrosis signaling pathway. **(B)** The potential influence on GM of BLM.

Next, HE and Masson staining, flow cytometry, qPCR, TUNEL staining, and IHC were used to verify and analyze the pathological changes of mice lung tissue to clarify the pulmonary toxicity mechanisms of BLM. The biological process related to PF induced by BLM included inflammation, fibrosis, and apoptosis. Our research confirmed that BLM could result in PF through the TGF-β/SMAD pathway to promote fibroblast and myofibroblast proliferation, increase the production of collagen, and cause the deposition of excessive ECM. Many cells make up the lungs, such as alveolar epitheliums, pulmonary macrophages, and fibroblasts. This study’s results found that massive cells of apoptosis existed in the lung tissues of the BLM group. Compared with the normal and sham groups, the apoptosis rate of pulmonary cells in the BLM group distinctly increased the expression levels of crucial target pro-apoptotic gene *caspase-3*, *BAX*, and the corresponding protein cleaved caspase-3 and BAX were apparently elevated. Hence, BLM may induce apoptosis of lung tissue cells such as alveolar epithelial cells. Therefore, anti-alveolar cell apoptosis might be a new curative direction for PF. The signal transduction of BLM PF in lung cells may mainly activate the caspase-3 apoptosis signaling pathway by inhibiting Bcl-2, activating caspase-8, BAX, caspase-9, and caspase-3, and inducing DNA breakage in the nucleus to produce apoptosis. At the same time, BLM activated the TGF-β/SMAD signaling pathway by promoting Smad2/3 to enter the nucleus, resulting in genes that promote fibrosis like *COL1A1*, *COL3A1*, *TGF*, *TGFBR1*, *fibronectin*, *α-SMA* expression. This will activate myofibroblast to secrete proteins that promoting fibrosis such as fibronectin, collagen III, and collagen I, resulting in a large ECM accumulation. The potential molecular mechanisms of BLM on PF in the caspase-3 apoptosis pathway and TGF-β/SMAD fibrosis pathway are shown in Figure 10A.

In addition, this study's results demonstrated that the relative abundance of some GM in PF mice changed significantly, indicating that PF disease will accompany a GM imbalance. Indeed, many bacteria within the microbiome are considered protective, such as *Lactobacillus* and *Catenibacterium.* The communication of host immunity and epitheliums with *Lactobacillus* plays a prominent role in the ecological performance of the intestine (Xiao et al., 2021). At the species level of *Lactobacillus* are probiotic-beneficial bacteria like *L. johnsonii* and *L. gasseri* (Kumar and Salminen., 2016). *L. johnsonii* has been proven to enhance the phagocytosis of peripheral blood leukocytes (Prakash et al., 2007). *Catenibacterium* is associated with the metabolism of short-chain fatty acid (Liu et al., 2021; Ma et al., 2021a) which regulates many segments, such as immunity/inflammation reaction and steady metabolism state, hormone secretion, cellular proliferation, and differentiation. *Verrucomicrobiales* can degrade complex carbohydrates (Mathai et al., 2021). In this study, mice of the BLM group with less diet lost weight, and a thin body may be associated with some carbohydrates degraded by increased *Verrucomicrobiales.* Some *Enterobacteriales* were listed in a preferential tabulation of pathogenesis bacteria by the WHO, some of which can give rise to extensive diseases in organisms (Taggar et al., 2020). Hence, we do not rule out the possibility that the decrease of probiotics *Lactobacillus* and *Catenibacterium* may be related to PF. Simultaneously, increased *Verrucomicrobiales* and *Enterobacteriales* may not be so friendly in the gut of PF mice. The potential influences on GM of BLM were shown in Figure 10B. In modern medicine, the lung and gut can be connected with each other through lymph (LouisMagnotti et al., 1998; Nakagaki et al., 2018; Ma et al., 2021b). Mesenteric lymph is characterized by anti-inflammatory and barrier protection. Mesenteric lymph takes part in the “gut-lung axis” of inflammation reaction. Gut-derived toxic factors invade the loop through the mesenteric lymph, reaching the pulmonary circulation and resulting in severe lung injury (Ma et al., 2021b). Therefore, it is supposed that, under the stimulation of PF caused by BLM, the intestine’s barrier function is also destroyed, changing the GM.

A growing body of research has identified the role of the GM in lung diseases such as COPD, asthma, lung cancer, respiratory infection, and PF (Chunxi et al., 2020; Zhan et al., 2021). Respiratory tract infection may be suppressed or prevented by adjusting the gut microorganism ecosystem (Qu et al., 2022). For instance, COPD is characterized by continuous airflow restriction, with the exact pathogenesis still unclear. The GM of COPD patients may be disturbed, along with decreasing GM diversity and immune system disorders, leading to chronic inflammation. Bowerman (Bowerman et al., 2020) reported that GM *Streptococcus sp000187445* and *S. vestibularis* negatively correlate with reduced lung function in COPD patients. Lai’s team (Lai et al., 2022) confirmed that COPD pathogenesis could be restored through fecal microbiota transplantation, and they also isolated a symbiotic bacterium, *Parabacteroides goldsteinii*, which proved to improve COPD. The significantly ameliorated mechanisms of COPD were alleviating gut inflammation, enhancing the activity of intestinal cell mitochondria and ribosomes, and inhibiting pulmonary inflammations (Lai et al., 2022). As for PF, Li’s research (Li et al., 2020) found that the PF mice model induced by irradiation of X-ray showed a similar trend between the lung microbiota and GM like *Alisipes*, *Lactococcus*, *Lactobacillus*, *Lachnoclostridium*, and *Bifidobacterium*. Among them, the abundances of lung microbiota *Alisipes*, *Lachnoclostridium*, and *Bacteroides* increased by irradiation, while there was a decrease of *Lactococcus*, *Dubosiella*, *Lactobacillus*, *Turicibacter*, *Candidatus-Saccharimonas*, *Romboutsia*, and *Bifidobacterium*. In GM after irradiation, there was an increase of *Alisipes*, *Mucispirillum*, *Helicobacter*, *Turibacter*, *Parabacteroides*, *Lachnoclostridium*, and *Intestinimonas*, while *Alloprevotella*, *Muribaculum*, *Anaerotruncus*, *Enterococcus*, *Bacteroides*, *Ruminiclostridium*, *Lactococcus*, and *Lactobacillus* decreased. Zhou et al. (2019) studied the GM of PF patients induced by silica and the results showed a decrease of *Bacteroides, Escherichia*, and *Shigella*, whereas those of *Megamonas*, *Lachnospiraceae*, *Lachnoclostridium*, and *Parabacteroides* increased. Therefore, PF will be accompanied by an imbalanced GM, just like some of the probiotics. However, many mysteries remain about the related mechanisms, which still require exploration.

In this study, there were differences in GM of *Lactobacillus* between BLM and normal mice, so we inferred that the specific *Lactobacillus* microbiota might have the potential to distinguish the two groups as biomarkers. The concept of the “gut-lung axis” offers a novel strategy for the follow-up clinical remedy of PF through the GM, which also tells us that intestinal health is not only related to intestinal health but also to lung health. We cannot thus regard PF as a single disease but need to start from the perspective of the whole organism to find more potential treatment mechanisms. The interposition of GM by regulating the “gut-lung axis” might be expected to treat respiratory-related diseases. Our research offers a new way of explaining the role of BLM in inducing PF from the perspective of GM. We hope this may provide new ideas and directions for dealing with PF in the clinic.

## Data Availability

The data presented in the study are deposited in the Sequence Read Archive (SRA) portal of NCBI repository, accession number PRJNA877383.
