# Supplementary material for: The gut-lung axis: Gut microbiota changes associated with pulmonary fibrosis in mouse models induced by bleomycin
Source: Front Pharmacol. 2022 Sep 30;13:985223. doi: 10.3389/fphar.2022.985223 (PMC9561135; doi:10.3389/fphar.2022.985223)
Supplement: Supplementary file 1 [file DataSheet1.docx]

Supplementary Material

# Supplementary Figures

This word file includes: Fig. S1 to S7.

## Supplementary Figures


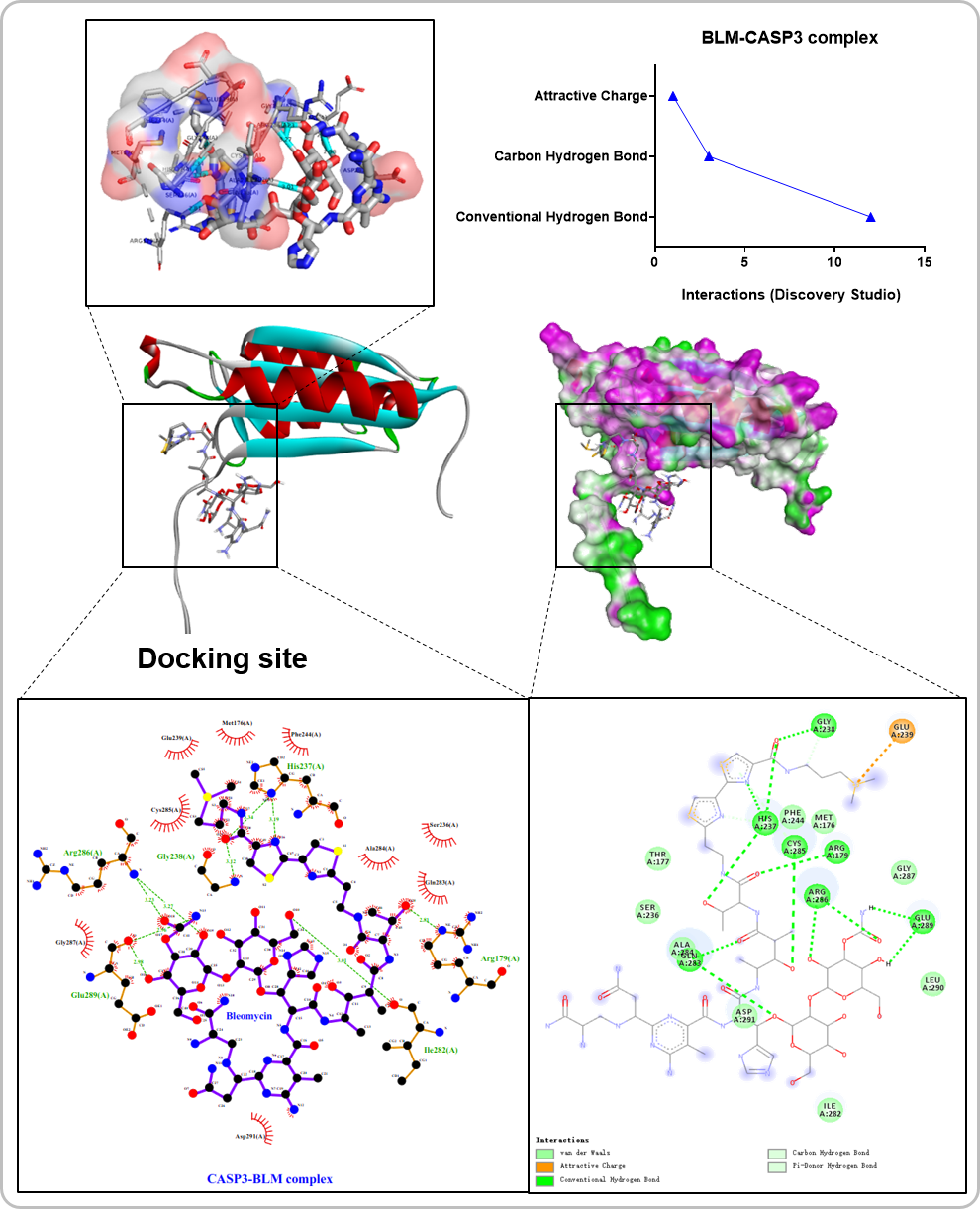


**Supplementary Figure 1.** **(Fig. S1.)** Visual analysis for BLM-CASP3 complex of molecular docking.


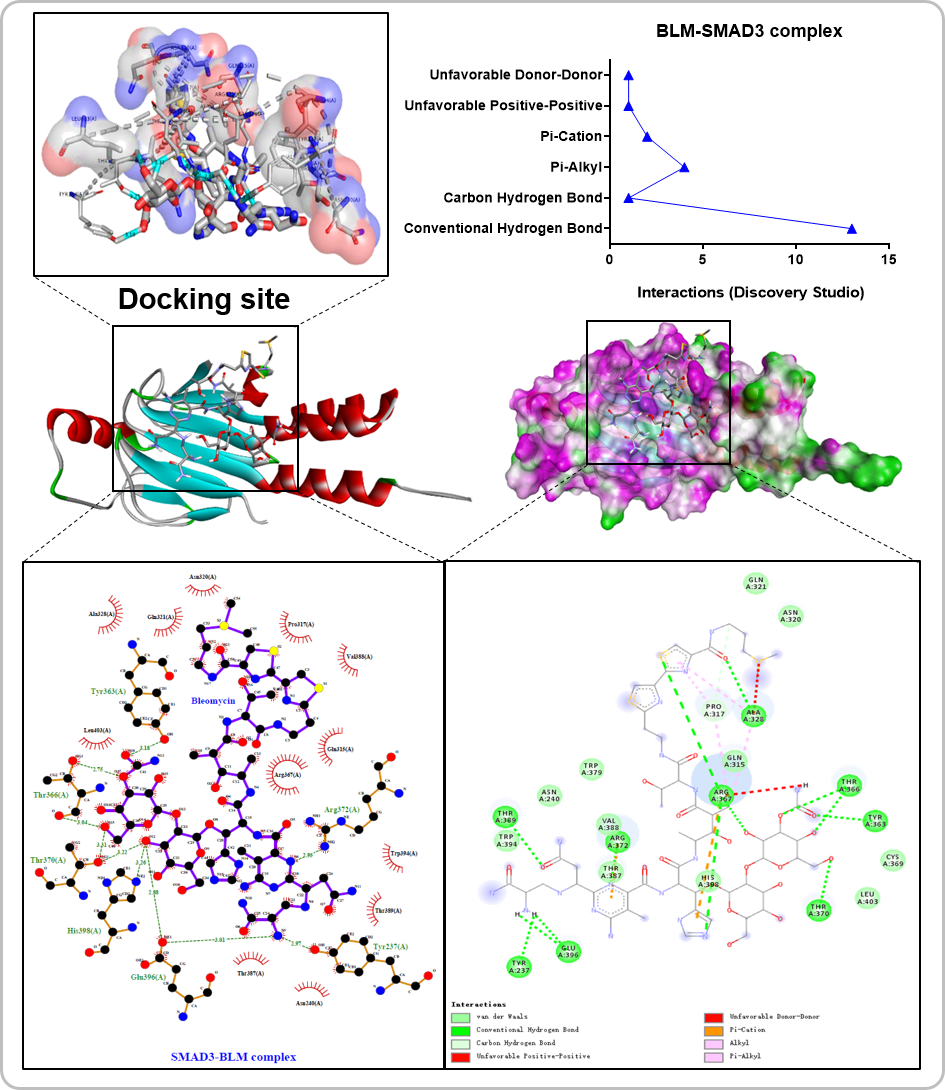


Supplementary Figure 2. (Fig. S2.) Visual analysis for BLM-SMAD3 complex of molecular docking.


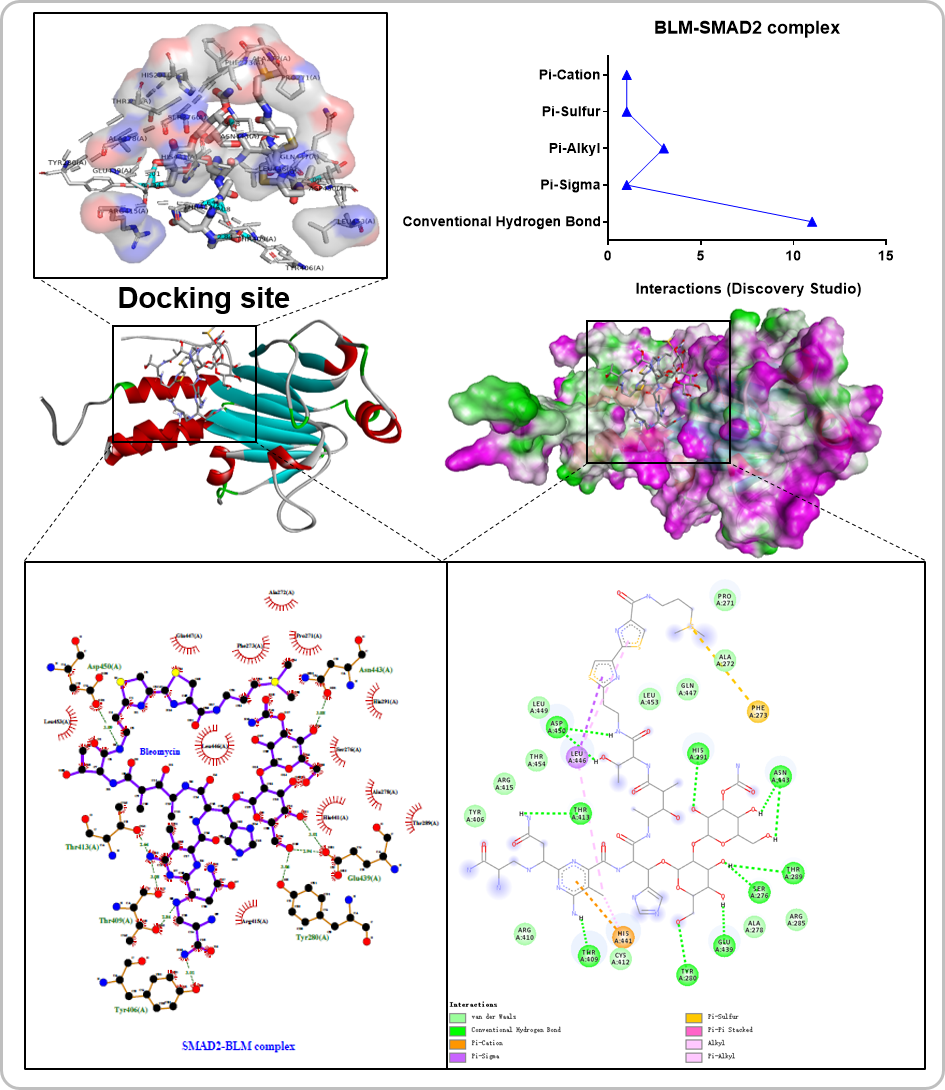


Supplementary Figure 3. (Fig. S3.) Visual analysis for BLM-SMAD2 complex of molecular docking.


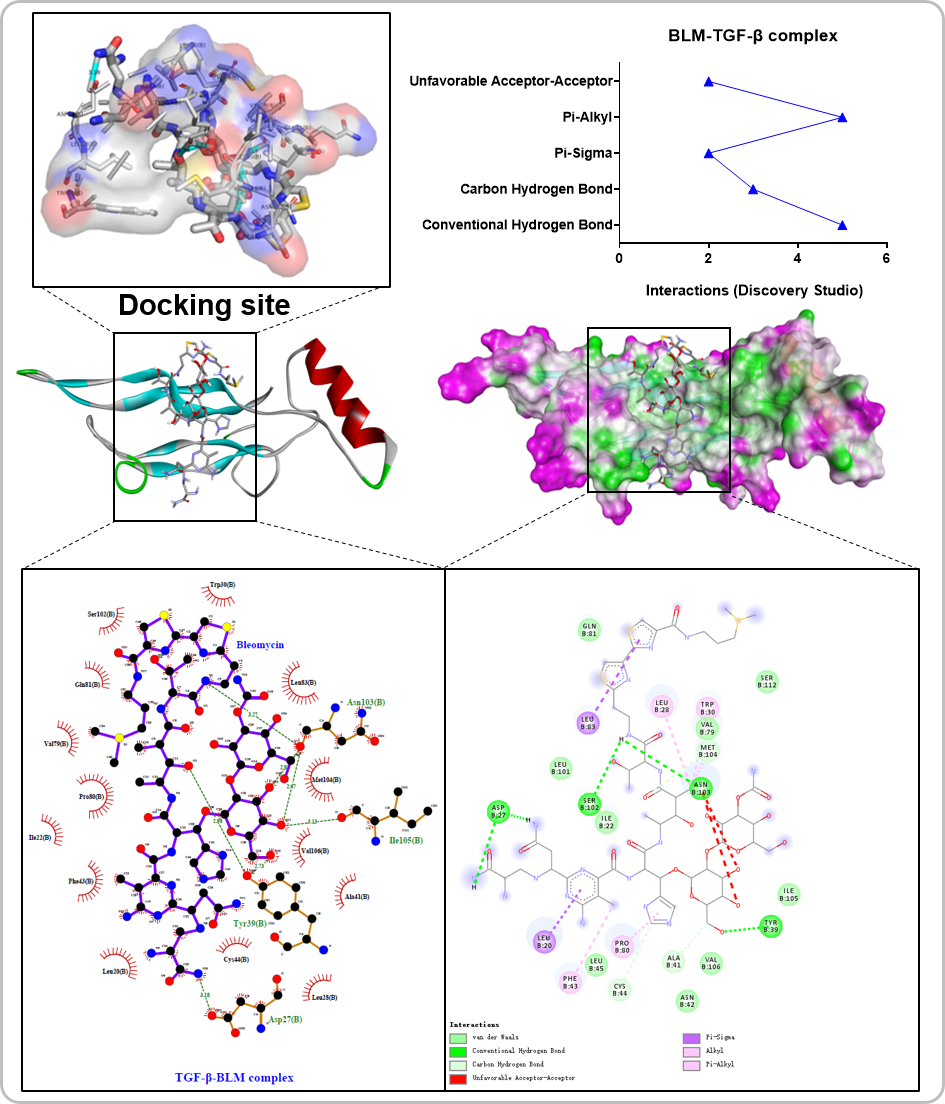


Supplementary Figure 4. (Fig. S4.) Visual analysis for BLM-TGF-β complex of molecular docking.


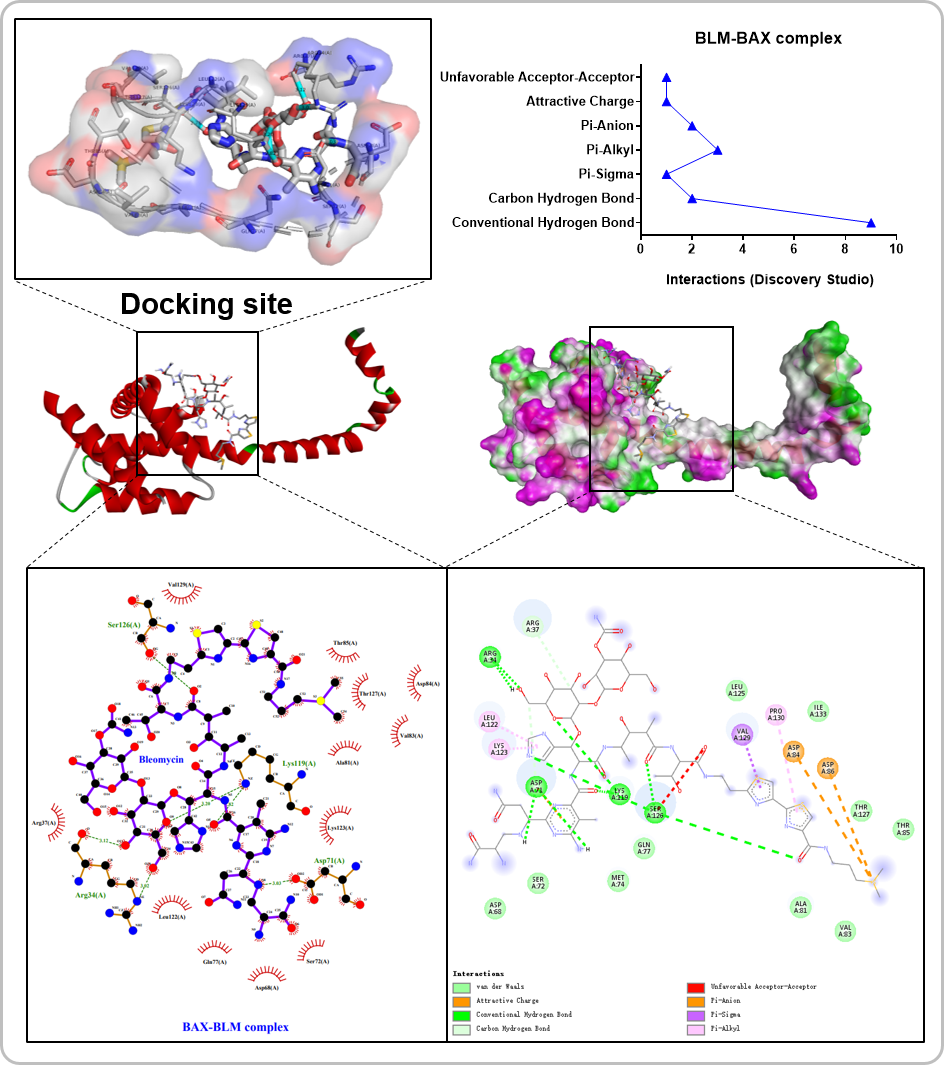


**Supplementary Figure 5. (Fig. S5.)** Visual analysis for BLM-BAX complex of molecular docking.


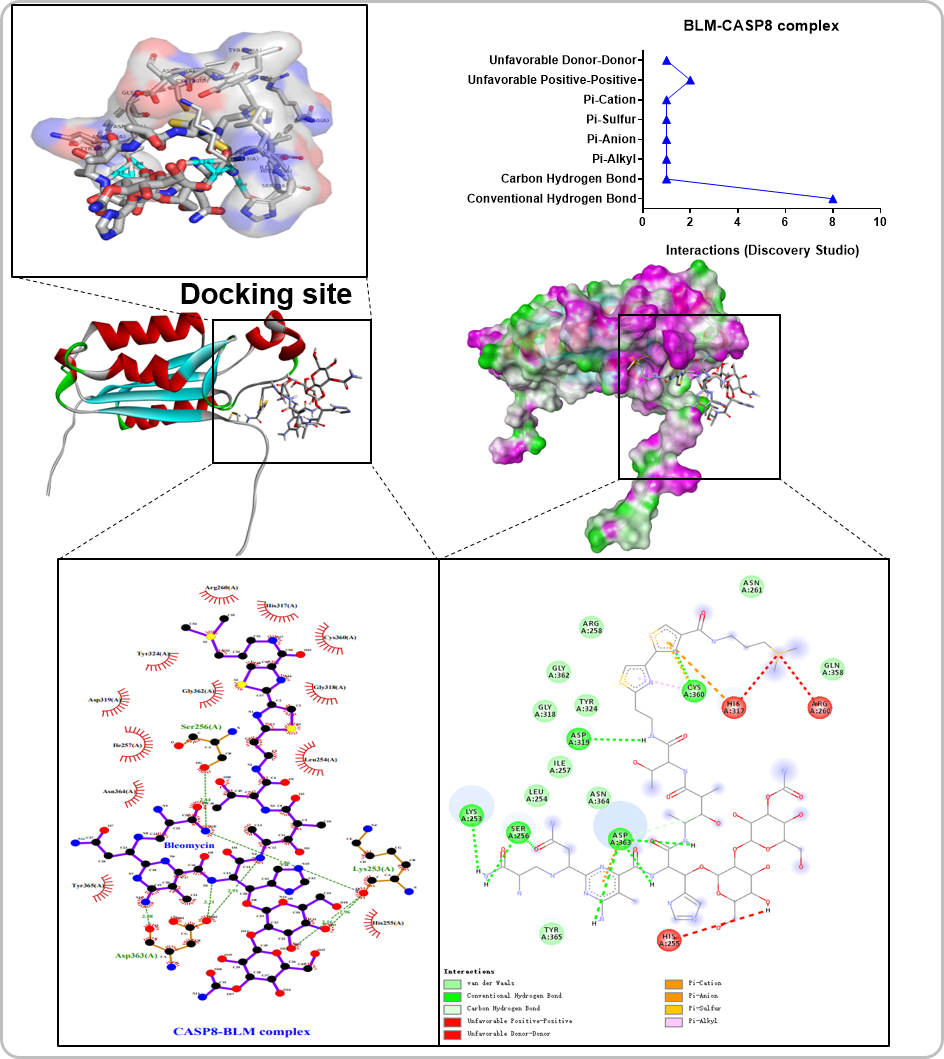


Supplementary Figure 6. (Fig. S6.) Visual analysis for BLM-CASP8 complex of molecular docking.


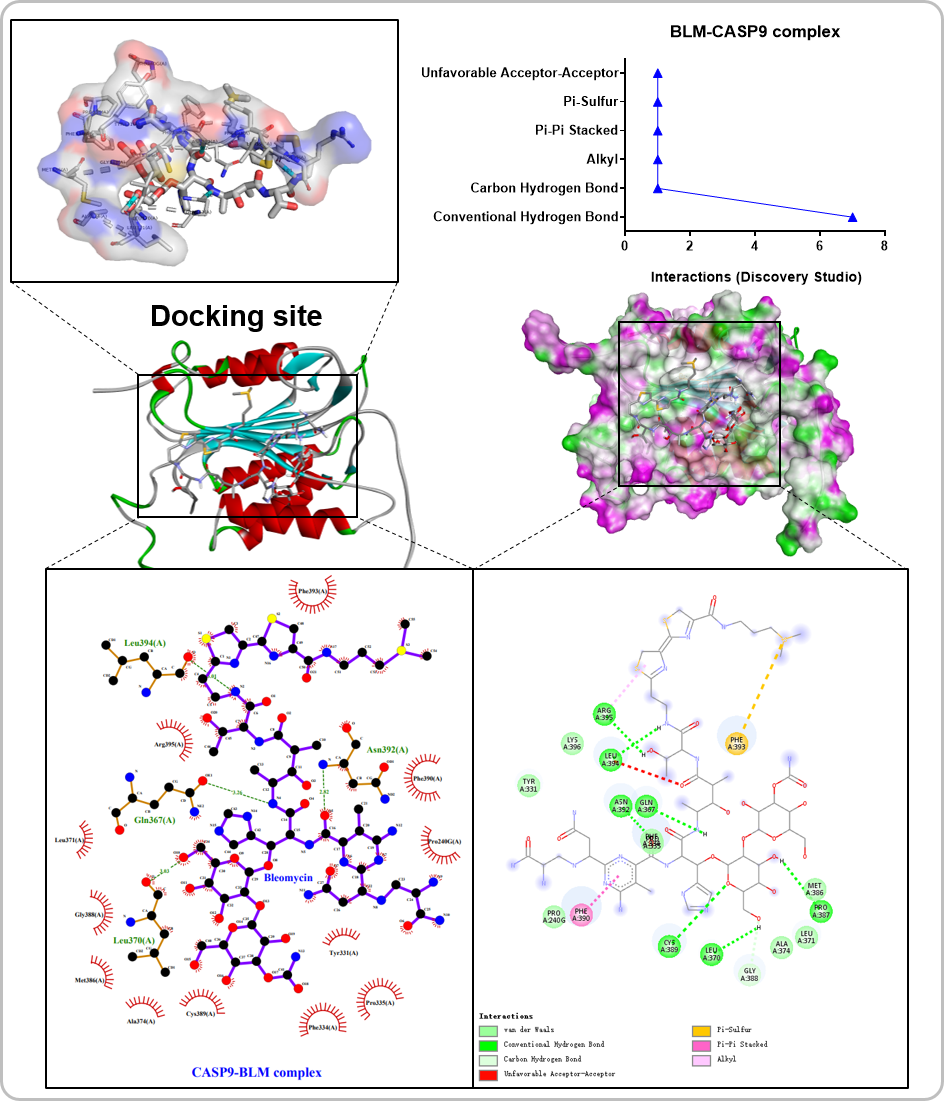


Supplementary Figure 7. (Fig. S7.) Visual analysis for BLM- CASP9 complex of molecular docking.
